# Therapeutic, and pharmacological prospects of nutmeg seed: A comprehensive review for novel drug potential insights

**DOI:** 10.1016/j.jsps.2024.102067

**Published:** 2024-04-16

**Authors:** Sawsan S. Al-Rawi, Ahmad Hamdy Ibrahim, Heshu Jalal Ahmed, Zhikal Omar Khudhur

**Affiliations:** a Department of Biology Education, Faculty of Education, Tishk International University, Erbil, KRG, Iraq; b Department of Pharmacy, Faculty of Pharmacy, Tishk International University, Erbil, KRG, Iraq

**Keywords:** Anticancer, Antidiabetics, Anti-inflammatory, Antimicrobial, Antioxidant, Bioactive compounds, Nutmeg, Oxidative stress, Psychotropics

## Abstract

**Background and objectives:**

For centuries, plant seed extracts have been widely used and valued for their benefits. They have been used in food, perfumes, aromatherapy, and traditional medicine. These natural products are renowned for their therapeutic properties and are commonly used in medicinal treatments. Their significant pharmacological profiles provide an excellent hallmark for the prevention or treatment of various diseases. In this study, we comprehensively evaluated the biological and pharmacological properties of nutmeg seeds and explored their efficacy in treating various illnesses.

**Method:**

Published articles in databases including Google Scholar, PubMed, Elsevier, Scopus, ScienceDirect, and Wiley, were analyzed using keywords related to nutmeg seed. The searched keywords were chemical compounds, antioxidants, anti-inflammatory, antibacterial, antifungal, antiviral, antidiabetic, anticancer properties, and their protective mechanisms in cardiovascular and Alzheimer’s diseases.

**Results & discussion:**

Nutmeg seeds have been reported to have potent antimicrobial properties against a wide range of various bacteria and fungi, thus showing potential for combating microbial infections and promoting overall health. Furthermore, nutmeg extract effectively reduces oxidative stress and inflammation by improving the body’s natural antioxidant defense mechanism. Nutmeg affected lipid peroxidation, reduced lipid oxidation, reduced low-density lipoprotein (LDL), and increased phospholipid and cholesterol excretion. In addition, nutmeg extract improves the modulation of cardiac metabolism, accelerates cardiac conductivity and ventricular contractility, and prevents cell apoptosis. This study elucidated the psychotropic, narcotic, antidepressant, and anxiogenic effects of nutmeg seeds and their potential as a pharmaceutical medicine. Notably, despite its sedative and toxic properties, nutmeg ingestion alone did not cause death or life-threatening effects within the dosage range of 20–80 g powder. However, chemical analysis of nutmeg extracts identified over 50 compounds, including flavonoids, alkaloids, and polyphenolic compounds, which exhibit antioxidant properties and can be used as phytomedicines. Moreover, the exceptional pharmacokinetics and bioavailability of nutmeg have been found different for different administration routes, yet, more clinical trials are still needed.

**Conclusion:**

Understanding the chemical composition and pharmacological properties of nutmeg holds promise for novel drug discovery and therapeutic advancements. Nutmeg seed offers therapeutic and novel drug prospects that can revolutionize medicine. By delving into their pharmacological properties, we can uncover the vast potential possibilities of this natural wonder.

## Graphical abstract

**Figure:**
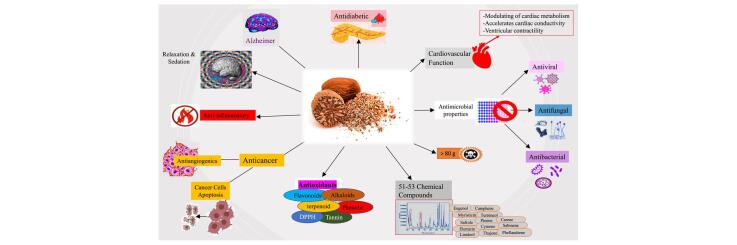
Unlocking the Potential: Exploring Therapeutic and Pharmacological Benefits of Nutmeg Seed

## 1 Introduction

Ensuring the safety of drugs and medicines is a major concern for pharmaceutical companies and researchers, who focus on dietary properties, toxicological consequences, and residual chemicals or purity. Moreover, the cost of modern medicine has increased significantly, making it unaffordable for 80 % of the population. Current therapies may have limited effectiveness and can cause side effects, resulting in pain and discomfort in many parts of the body (Al-Rawi et al., 2023). Ensuring the safety and affordability of medical treatments is currently a top research priority (Dogara et al., 2022). The use of traditional medicinal plants dates back 4000 years to Mesopotamia, where seeds and plant extracts have been used to prevent or cure diseases, as described in clay tablets (Dahham et al., 2018). Plants and seeds possess an array of secondary metabolites and aromatic substances that make them valuable resources for the treatment or prevention of various illnesses. Their extracts contain compounds that can be used either alone or combined with other therapeutic agents to enhance their effectiveness and reduce the risk of medication-related side effects. Their use is considered safe and has been widely adopted in medical practice (Ibrahim et al., 2011). Nutmeg seed has been used in traditional medicine by most civilizations since prehistoric times. From an ethnobotanical point of view, nutmeg seeds have been utilized extensively by various cultures in cooking and fumigating due to their strong scented aroma and medicinal properties. Beyond its medicinal value, nutmeg played a prominent role in many societies during ceremonies and rituals, which reflects its cultural versatility. Nutmeg seed has been also used in traditional remedies because of its therapeutic properties, such as stomach pain, ache relief, aphrodisiacal and Abortifacient (Tripathi & Dwivedi, 2015). Nutmeg seeds have been used as spices in many countries because of their appealing pleasing aroma and sweet flavor (Al-Rawi,et al., 2011). The best description of nutmeg, as quoted by Nagano, (2009) “Nutmeg is a well-rounded little nut. It may be used to brighten your day, spice up your love life, to flavor your food, to induce vivid dreams, or to just get plain stoned. This seed has been overlooked and misunderstood by many ethnophiles, but once one is privy to her secrets, she can become a valuable all”. Over the years, numerous studies have been conducted on the qualities and chemical components of nutmeg extracts. These extracts, along with the essential oil derived from nutmeg, are widely used in the food industry as well as in the development of pharmaceutical and medicinal products. The versatility of nutmeg makes it an important ingredient in various fields, including culinary arts, healthcare, and pharmaceuticals. (Ibrahim et al., 2020). Nutmeg seed has various pharmacological effects, antidiarrheal, and analgesic effects, with a significant sedative property (Grover et al., 2002). It has been extensively studied, and it has been found to contain a range of bioactive chemical compounds, including volatile oils, phenolic compounds, alkaloids, and flavonoids, (Spricigo et al., 1999, Rancy and Krishnakumari, 2015). Nutmeg also possesses numerous potential compounds such as myristicin, eugenol, elemicin, safrole γ-terpinol, and α-pinene (Matulyte et al., 2019; Ibrahim et al., 2018). These compounds exhibit various pharmacological traits, such as anti-inflammatory, anticancer, and neuroprotective effects (Zhang et al., 2016, Lee et al., 2011, Liu et al., 2022). However, because of its poisonous effects, nutmeg has been overlooked, misunderstood, and underestimated, with a quantity of 5 g can be harmful and poisonous (Hallström, 1997). There is a contradiction in the existing literature regarding the safety and efficacy of nutmeg seeds as a therapeutic agent, which highlights the need to further elucidate their pharmacological activity. Nutmeg seeds have significant therapeutic potential, particularly for the development of novel drugs. Despite its promising therapeutic potential, yet some voids still exist and need to be filled by reviewing the recent finding behind its mechanism of action of nutmeg. Nutmeg seed possess exceptional pharmacokinetic characteristics, due to its active compounds metabolites such as myristicin, safrole, and elemicin (Yang et al., 2015, Song et al., 2019). Several pathways of metabolism have been suggested, including hydroxylation, demethylenation, and amination of the allyl group (Neukamm et al., 2022). However, a deeper understanding of the pharmacokinetics of nutmeg, and its active compounds interaction within the body system will help in filling the gap and can offer new insight in developing safe and effective nutmeg-based drugs. Moreover, the metabolizing and excretion mechanism of nutmeg whether in vivo or in vitro could pave the way for future application in modern medicines. Thus, this review aims to rigorously evaluate the existing publications, identify gaps, and offer insight into the potential role of nutmeg as an alternative or complementary therapy in the treatment of various diseases. An in-depth analysis of the effectiveness of nutmeg seeds and their therapeutic properties was conducted, such as nutmeg chemical composition, antioxidant, antimicrobial, anticancer, antiangiogenic, and its application in treating chronic diseases. In addition, the pharmacokinetics, bioavailability of nutmeg, and its recent application in clinical trials will be elucidated further to understand its potential therapeutic approach. Overall, unlocking the therapeutic potential of nutmeg seed can pave the way for its application in several areas to significantly impact both industries and people's well-being. Additionally, the findings of this study address a potential increasing interest in natural medicines, which can offer new insights into new therapeutic novelties for countless health conditions. Such innovation has potential for pharmaceutical, nutraceutical, and food industries, which can lead to the advancement of new drugs and nutmeg-based products.

## 2 Methodology

A thorough search was conducted across a range of databases, including Google Scholar, PubMed, Scopus, Web of Science, ScienceDirect, and Wiley. This was done to ensure the inclusion of a diverse range of articles to achieve a comprehensive understanding of the topic under investigation. The search was conducted meticulously with a focus on accuracy and completeness. This meticulous keyword search aimed to identify relevant articles related to nutmeg seed extraction and its pharmacological properties. Published articles on nutmeg seed extraction until 2023 were searched across the aforementioned databases. During the search, a variety of keywords were used to find relevant information on nutmeg, including descriptions, taxonomy, history, traditional and modern uses, extraction methods, chemical composition identification, anti-inflammatory, antioxidant, antifungal, antibacterial, antiviral, and anticancer, psychotropics and toxicological properties. We also looked for data on its pharmacokinetics, bioavailability, and the application of nutmeg in treating chronic diseases, such as diabetes, cardiovascular disease, anxiety, depression, Alzheimer's disease, and in clinical trials. However, we excluded articles that did not meet the inclusion criteria, such as those not in English, theses, unreliable online sources, *meta*-analysis, and systematic reviews articles, or those that primarily focused on molecular docking.

## 3 Results and discussion

Initially, approximately 2,140 articles with relevant titles were identified through a meticulous keyword search. Following this, duplicate articles were removed, reducing the number to 1,360 articles. Subsequently, a rigorous filtering process was applied to exclude any irrelevant articles, ultimately narrowing the total number to 235. This approach ensured that the articles included in the study were comprehensive and focused. These articles were sought to investigate the pharmacological properties of nutmeg seeds in treating various chronic diseases and illnesses, as well as its pharmacokinetics, bioavailability and its investigation in clinical trials. The data collected from the selected articles are highlighted and organized accordingly, either in tables or in text form. The collected data confirmed that the origin of nutmeg can affect its chemical composition as well as its pharmacological activities, as shown in Table 3. Despite the different extraction methods, however, the chemical compounds were almost the same, yet varied in concentration and in the number of constituents, which affected the pharmacological and therapeutic properties of nutmeg extracts. The wide application of nutmeg as a flavoring agent and functional food product has gone far beyond this. Its attraction lies in its major or main compounds that have antioxidant properties, which make it a good candidate for future novel drugs for therapeutic purposes. Based on the identified chemical components that are present in nutmeg, the chemical structures of these components were obtained from the National Institute of Standards and Technology, 2023 (NIST) as shown in Fig. 3. However, the explored collected data are presented and discussed in detail in the following texts, to elucidate the significant characteristics of nutmeg that make it worthy of further research.

### 3.1 History and importance of nutmeg seed

The nutmeg is the seed of the Myristica *fragrance* fruit that has a yellow peachlike shape and grows from *Myristica fragrans* tree. The tree is an evergreen, aromatic dioecious tree commonly cultivated in tropical regions, particularly Southeast Asian countries, tropical America, and the Pacific Islands. It originated from the Banda Island of Indonesia, which is called the Maluku or the Spice Islands. Historically, the Arab transferred the nutmeg into Europe until the discovery of the nutmeg trees by the Portuguese on Banda Island in the 15th century (Payne, 1963). Later on, during the 17th century, when the Dutch colonized the Spice Islands, and they maintained control over the trade in spices, and until the end of the 18th century, when the British acquired nutmeg seedlings from the Banda Islands (Barceloux, 2009). Recently, there has been a high demand for nutmeg seeds in developed countries. Germany, Japan, the United States, and Europe are among the top nutmeg seed importers. Several countries, including Indonesia, India, Sri Lanka, and Grenada, are well known for their exporting of nutmeg (Purba et al., 2021, Gordon, 2020, Private Sector CARICOM’s nutmeg trade, 2009). Currently, the demand for high-quality nutmeg is on the rise owing to its significant value in the baked goods, pharmaceutical, and cosmetics industries. The intriguing blend of fixed oil, essential oil, and oleoresin found in nutmeg seed extract makes it a desirable industrial product in food and pharmaceutical products. The oil of the nutmeg seeds and its derivatives are widely used as flavoring additives in various food products, whereas the nutmeg oleoresin is a rival to the dry seeds. The fixed oil makes up roughly 20–40 % and is extremely aromatic, accounting for 8–15 % of the mixture. Thus, nutmeg oil production can replace the dried seeds because this product is highly aromatic and free of aflatoxin (FAO, 1994).

### 3.2 Nutmeg seed in traditional and Ayurveda medicine

Myristica fragrans, commonly known as nutmeg, is a highly desired spice that has been used since ancient times as a remedy for many illnesses as well as in aromatherapy. Nutmeg has been widely used in traditional Indian medical science, and Ayurveda. In the seventeenth century, physicians used nutmeg pomander to treat the black plague (Freedman, 2015). Nutmeg is also mentioned in Unani medicine for regulating sexual disorders (Ahmad et al., 2005). Additionally, nutmeg has been used in traditional medicine across all Asian countries. Nutmeg seeds have been used to treat various ailments such as stomachache, dysentery, nausea, rheumatism, vomiting, malaria, and sciatica (Ogawa & Ito, 2019). Nutmeg was also used as an appetite stimulant, tonic, carminative, aphrodisiac, and electuaries for leprosy. It was also used to treat intestinal catarrh, heartburn, gases, and for menstruation and as an abortifacient (Okiki, et al., 2023). In the past decade, pregnant women used to consume large amounts of nutmeg to abort their babies (Smith, 2014). The use of nutmeg was reported in the Medical Book of Malayan Medicine as a remedy for overeating, bloating, malaria, and madness (Weil, 1971). Apart from that, nutmeg oil and butter have traditionally been applied as topical treatments for ailments such as headaches, rheumatism, and sprains. It has been suggested that adding a drop of nutmeg oil to a cup of tea can help with indigestion and vomiting (Gils & Cox, 1994). In Indonesia, nutmeg is still being used to treat vomiting, stomach, rheumatism, and kidney disorders, as well as to alleviate stomach cramps, nervousness, and whooping cough. Nutmeg is a key ingredient in JamuBeraskencur, which is a herbal drink traditionally used in Indonesia to treat stiffness and pain (Sumarni et al., 2019). In Asia, nutmeg was used as a post-childbirth tonic in Malay medicine (Van Gils & Cox, 1994). Nutmeg has been traditionally used in Africa and Nigeria to treat digestive and respiratory tract illnesses, such as stomach cramps, diarrhea, and respiratory discharge (Okiki, et al., 2023). On the other hand, in Western countries, Europe, the Middle East, and Central America, nutmeg is usually used as a spice in desserts and many dishes in addition to other countries around the world like Asia and Africa (Loizzo et al., 2016).

### 3.3 Nutmeg seed in industries

Nutmeg and its oleoresin are widely used in different industrial production (Eweka and Eweka, 2010). The Nutmeg fixed oils, essential, and its fatty products are widely used in pharmaceutical products, cough syrups, soap industries, cosmetics, and balms. In addition, nutmeg is widely used in food industries, while the primary uses of nutmeg oil and oleoresin are in culinary applications including sauces, soups, spice blends, processed meat, preserves, cheese, baked goods, desserts, and egg dishes (Morsy, 2016). Therefore, there is a growing market for nutmeg-based products, oils, and oleoresins as a seed substitute, because they are clean and free from aflatoxin (ITC, 2003).

### 3.4 Taxonomic classification and overview of nutmeg

**Fig. 1:**
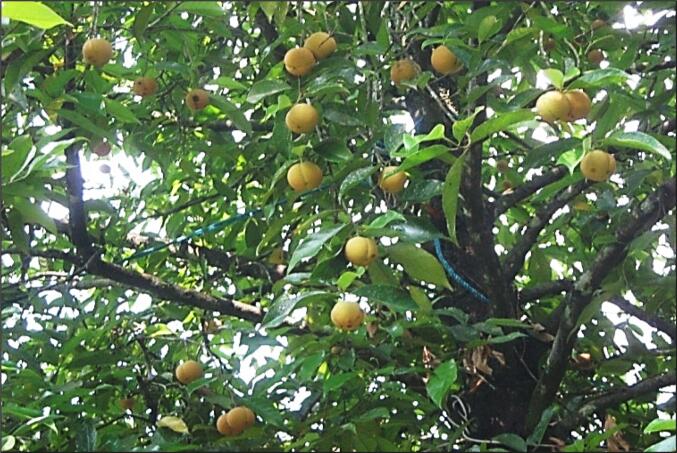
Nutmeg (Myristica fragrans) tree from Balik Pulau, Pinang. (Author Source).

*Myristica fragrans* is the scientific name of nutmeg that belongs to the Myristicaceae family. It is known by various common names or synonyms. It is known as mace or nutmeg in the United Kingdom, bunga pala in Indonesia, nuez moscada in Germany, muscadier in France, jaiphal in India, Muskatbaum in Uruguay, or Spain, and jawzat altayib in Arabic countries. The nutmeg taxonomic classification obtained from the Integrated Taxonomic Information System (ITIS, 2023) is presented in Table 1. The *M. fragrans* tree is commonly cultivated in Southeast Asia and several other countries, including Indonesia, Malaysia, Grenada, Sri Lanka, India, and Vietnam (Olaleye et al., 2006, Pham et al., 2000, Gils and Cox, 1994, Al-Rawi et al., 2013). The tree is originally from the Spice Islands in Indonesia, also known as the Banda Islands. These tropical, aromatic evergreen trees are 9–12 m tall and have scattered branches. The tree is dioecious, meaning that it has separate male and female flowers occurring on the same tree. The leaves are dark green, arranged alternately along the branches, and range from 5 to 15 cm in length and 2 to 7 cm in width. The flowers are waxy, fleshy, bell-shaped, and light yellow in color. The tree starts producing fruit after six years and can continue producing year-round for 20–75 years (ITC, 2003, Barceloux, 2009). The fruit is fleshy, green, or yellow in color, and similar to an apricot or peach in appearance (Fig. 1). When it ripens, the fruit splits into two halves, to reveal a shiny purplish brownie seed enclosed by a scarlet aril, which is called Mace.

**Table 1:** Shows the taxonomic classification of *Myristica fragrans* Houtt. (nutmeg). Obtained from the Integrated Taxonomic Information System (ITIS, 2023).

Kingdom	Plantae – Plantae
Subkingdom	Tracheobionta
Phylum	Tracheophyta
Super division	Spermatophyta – Seeds
Division	Magnoliophyta
Class	Magnoliopsida
Subclass	Magnoliidae
Order	Magnoliales
Family	Myristicaceae
Genus	Myristica Gronov.
Species	Myristica fragrans Houtt.

### 3.5 Nutmeg seed description

**Fig. 2:**
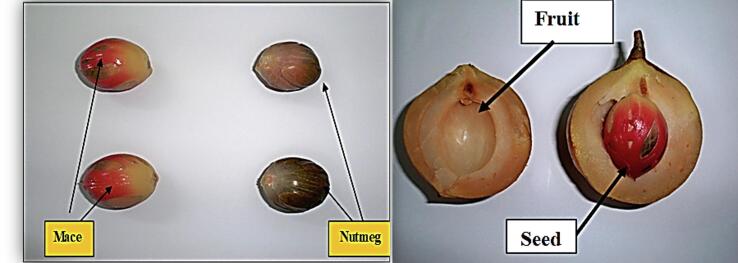
Right image; shows a close up to a nut of Myristica fragrans fruit. Left image shows the nutmeg seed, and mace (author Source).

Nutmeg, the dried seed of *Myristica fragrans*, has a distinctive pleasant fragrance and sweet flavor. The seed has an elongated oval shape with a wrinkled surface and a light brown color. The ripe seed of nutmeg has a whitish color, is fleshy, firm, crossed diagonally by red-brown strains, and is surrounded by a fleshy bright red cover (scarlet aril), which is known as mace (Fig. 2). The nutmeg seeds and maces are treated separately after being dried and used as spices.

### 3.6 Composition of nutmeg seed (*Myristica fragrans*)

Understanding the composition of an oil offers considerate information about its possible applications in many areas. It also provides a better understanding of its role in health and its use as a treatment for many diseases. Raw nutmeg seeds contain 30 %–55 % oils and 45–60 % solid matter, including cellulose. The nutmeg oil comprises a crude fixed oil known as nutmeg butter, which makes up 20–40 %, and an essential oil, which accounts for 8 %–15 %, as well as other oleoresins. This nutmeg butter is highly aromatic owing to the presence of an aromatic group (Parthasarathy et al., 2008). Nutmeg also contains protein, lipids, and starches (Rahardiyan, et al., 2020). Nutmeg oleoresin has a strong and distinctive scent and flavor, which makes it a popular alternative to dry nutmeg seeds in various industries such as pharmaceuticals and food (ITC, 2003). Thus, the butter, essential oil, and oleoresin of nutmeg are considered excellent substitutes for whole nutmeg and can be widely used in the market. In general, the common extraction method for fixed oil is by applying pressure and heat using hydraulic extraction or via Soxhlet extraction. Both methods produce an aromatic orange extract with a semi-solid texture, which is called nutmeg butter. The extraction method has a great influence on the chemical constituents of the yield and essential oils (Ibrahim and Al-Rawi, 2018, Matulyte et al., 2019). Soxhlet extraction of nutmeg yielded 34 %, whereas supercritical extraction yielded 38.8 % (Al-Rawi, et al., 2013). In contrast, the essential oil of nutmeg is extracted by steam distillation. The finished product has a nutmeg flavor and aroma and is either a colorless oil or a pale-yellow liquid (Umayah and Marhaendro, 2021). It is soluble in alcohol but insoluble in water, sensitive to light and air, and thus must be stored in a closed container.

### 3.7 Chemical properties of nutmeg seed

Characterization of the chemical constituents present in an extract is essential for confirmation and standardization (Radzali et al., 2022). The chemical components of nutmeg were identified using various identification techniques, including GC and GCMS, HPLC, and GCTOFMS. Ibrahim and Al-Rawi (1918) reported the chemical composition of supercritical nutmeg extract using GC–TOFMS. Over the last few years, more research has been conducted on nutmeg essential oil than on butter or fixed oil. Despite having a lower concentration than the fixed oil found in nutmeg seeds, its popularity grew significantly. This is most likely because, in addition to its other pharmacological qualities, essential oil has the necessary valuable components for industrial products. The main ingredients of nutmeg essential oil have been documented in several studies (Kapoor et al., 2013, Yuan et al., 2006, Ginting et al., 2017, García-Díez et al., 2017, Maya et al., 2004). Muchtaridi et al. reported the chemical makeup of nutmeg seed essential oil. The chemical and physical properties of Sri Lankan nutmeg oil have also been reported by Subaddarage et al. (1985). However, the quality of nutmeg extracts can be assessed on the basis of the presence and concentration of these chemical constituents. In general, there are six major groups of chemical compounds present in nutmeg (FAO, 1999), as shown in Table 2.

**Table 2:** The six major groups present in nutmeg (FAO, 1999).

1. Aromatic ethers: this group comprises the following: myristicin, safrole, eugenol, methyl *iso*-eugenol, methoxy eugenol, *iso*-eugenol, methyl eugenol, elemicin, and *iso*-elemicin.
2. Terpene group: this group comprises the following: Alpha and gamma-terpinene, terpinolene, alpha and beta-pinene, alpha and beta-phellandrene, alpha-thujene, delta3-carene, myrcene, camphene, uinonene, dipentene, and sabinene.
3. Monoterpene Alcohol: this group comprises the following: Alpha-terpineol, beta-terpineol, geraniol, citronellol, terpineol, caryophyllene, sesquiterpene, and linalool.
4. Terpinic esters; this group comprises the following: Bornyl acetate, Geranyl acetate, and Linalyl acetate.
5. Aromatic hydrocarbons: this group comprises the following: toluene and p-cymene.
6. Acids: this group comprises the following: octanoic, butyric, formic, and acetic acid.

**Table 3:** Illustrates the chemical composition of nutmeg seed extracts from different geographical regions, which were extracted using different extraction methods.

Compound	Method of extraction	Identification method	Composition %	Reference
Myrislignan	Grenada nutmeg seed extracted by Maceration	GC–MS	22.59	Matulyte et al., 2019
Elemicin			13.99	
α-Phellandrene			13.04	
Isomethyleugenol			6.38	
β-Myrcene			4.6	
4-Carene, trans			3.37	
Sylvestrene			1.57	
Isogermacrene			1.61	
γ-Asarone			0.79	
Cis-p-menth-2-en-1-ol			0.43	
Myristic a*cid*	Local market nutmeg seeds extracted by Supercritical fractioned carbon dioxide extraction	HPLC-DAD	79.2 %	Piras et al., 2012
Myristicin			32.8 %	
Sabinene			16.1 %	
Alfa-pinene			9.8 %	
βeta-pinene			9.4 %	
Oleic acid			7.4 %	
Palmitic acid			6.1 %	
Terpinen-4-ol			3.6 %	
β-phellandrene			4.9 %	
Safrole			4.1 %	
	
Sabinene	Grenada nutmeg seeds extracted by Hydrodistillation	GC–MS	52.75	Mickus et al., 2021
α-pinene			13.53	
D-limonene			6.96	
α-terpinyl acetate			5.98	
β- pinene			3.58	
γ-terpinene			3.31	
β- myrcene			2.91	
α-phellandrene			0.47	
Myristicin			1.88	
α-thujene			1.81	
Germacrene D			1.00	
α-terpinene			0.91	
α-copaene			0.8	
4-thujanol			0.75	
Cis-p-menth-2-en-1-ol			0.15	
Bornyl acetate			0.15	
α-terpinolene			0.14	
γ-amorphene			0.14	
Bicyclogermacrene			0.12	
β-cubebene			0.2	
Camphene			0.2	
Isogermacrene D			0.07	
β-caryophyllen			0.07	
Cis-α-bergamotene			0.09	
	
Sabinene	Indian nutmeg seeds extracted by n-hexane extraction method	GC–MS	12.2	Hoda et al., 2020
Oleic acid			11.7	
Hexadecanoic acid			10.5	
Safrole			8.1	
Elemicin			7.8	
Linoleic acid			6.9	
Myristicin			6.7	
*β*-Pinene			6.5	
Limonene			4.5	
*α*-Pinene			3.8	
*α*-Phellandrene			1.8	
Myrcene			1.7	
Terpinolene			1.5	
*α*-Thujene			1.4	
Methyl eugenol			1.3	
*γ*-Terpinene			1.1	
*α*-Terpinene			0.8	
*trans*-Sabinene hydrate			0.8	
Terpin-4-ol			0.8	
*α*-Humulene			0.7	
*cis*-Sabinene hydrate			0.7	
*δ*-3-Carene			0.5	
(*E*)-Methyl isoeugenol			0.8	
*β*-Caryophyllene			0.7	
(*E*)-Isoeugenol			0.5	
(*E*)-Isoelemicin			0.4	
Germacrene D			0.4	
Ethyl hexadecanoate			0.4	
*δ*-Elemene			0.3	
*α*-Terpineol			0.3	
*α*-Copaene			0.3	
13-*epi*-manool oxide			0.3	
α-Fenchene			0.3	
*o*-Cymene			0.3	
1,8-Cineole			0.2	
Kaurene			0.1	
1,3,8-p-Menthatriene			trace	
*β*-Cubebene			t	
*γ*-Muurolene			t	
Sabinene hydrate acetate			t	
Eugenol			t	
Camphene			t	
	
Gamma-terpinene	Indian nutmeg seeds extracted by n-hexane extraction method	GC–MS	1.43	Al-Qahtani et al., 2022
Thymol			1.06	
Alpha-terpineol			0.76	
Alfa-Copaene			0.88	
Eugenol			2.68	
Safrole			2.40	
Methyl eugenol			3.82	
Caryophyllene			1.51	
Trans-Isoeugenol			2.61	
Isoelemicin			4.09	
Myristic acid			22.25	
Phenol, 2,6-dimethoxy-4-(2-propenyl)-			2.97	
Elemicin			24.44	
Myristicine			13.81	
Naphthalene,			0.91	
Methyl isoeugenol			10.84	
Anethole			0.95	
3-Cyclohexe, 1-ol-4-methyl-1-(1-methyl ethyl)-(R)-			2.92	
Cyclohexanol, 1-methyl-4-(1-methylethenyl)-cis-			1.27	
Cyclohexanol, 1-methyl-4-(1-methyl ethyl)-			0.93	
Sabinene	Nutmeg seed produced in Guangdong, China Extracted By Steam Distillation	GCMS	25	Zhang et al., 2016
α-Pinene			12.79	
4-Terpineol			11.54	
α-Thujene			2.15	
Limonene			6.87	
γ-Terpinene			6.52	
α-Myrcena			1.98	
α-Terpinene			4.28	
Safrole			3.07	
Terpinolene			2.45	
Myristicin			2.4	
Elimicin			1.2	
*trans*-Sabinene hydrate			0.12	
Linalool			0.35	
Camphene			0.27	
α-Terpineol			0.87	
Bornyl acetate			0.17	
*trans*-β-Ocimene			0.04	
Citronellol			0.14	
Isoeugenol			0.09	
Methyl eugenol			0.74	
*cis*-Sabinene hydrate			0.08	
Myristicin	Indonesia nutmeg seeds extracted by 2 steps steam distillation. 12 h distilled steam without pressure and 12 h distilled steam using pressure.	GCMS	30.3	Umayah & Marhaendro, 2021
α.-Pinene			12.01	
4-Terpineol			9.75	
β.-Pinene			9.65	
Terpinene			6.63	
Limonene			4.99	
Phellandrene			4.61	
Carene			4.41	
Safrole			2.76	
α.-Terpinolene			1.99	
α.-Thujene			1.94	
α.-Terpineol			1.83	
β.-Myrcene			1.82	
Eugenol			0.92	
Isoeugenol			0.9	
Phellandrene			0.89	
Asarone			0.86	
Pentylanisole			0.77	
Neryl acetate			0.74	
α.-Copaene			0.64	
δ-3-Carene			0.41	
p-Cymene			0.39	
Methyleugenol			0.39	
Camphene			0.21	
α.-Terpinyl acetate			0.19	
Myristicin	Indonesia nutmeg seeds extracted by steam distillation the extraction time was 180 min.	GC/MS and GC/FID methods,	4.0	Nikolic et al., 2021
α-Thujene			0.9	
α-Pinene			5.73	
Sabinene			42.3	
Myrcene			2.7	
α-Phellandrene			0.6	
δ-3-Carene			0.6	
α-Terpinene			1.3	
p-Cymene			0.6	
Limonene			6.4	
β-Phellandrene				
γ-Terpinene			2.6	
Cis-Sabinene hydrate			1.0	
Terpinolene			1.2	
Linalool			1.3	
dehydro-Sabina ketone			Trace	
Terpinen-4-ol			6.3	
α-Terpineol			0.7	
Safrole			1.3	
α-Copaene			0.5	
Methyl eugenol			8.0	
(E)-Caryophyllene			0.7	
Germacrene D			0.6	
(E)-Methyl isoeugenol			3.0	
sulfonylbismethane	China nutmeg seed extracted with ETHANOL EXTRACTION	GCMS	27.22	Wagan et al., 2017
Alpha-phellandrene			0.13	
Delta-3-carene			0.03	
Sabinene			0.53	
Beta-pinene			0.12	
Gamma-terpinene			0.33	
Trans-sabinene hydrate			0.28	
4-oxo-beta-isodamasco			0.31	
Methenocyclopentapyrazole			0.95	
1,3-benzodioxole			0.78	
Alpha-cubebene			4.84	
Methoxy-isoquinolin-6-ol			1.86	
4-methoxy-1,3-benzodioxole			7.29	
1-methoxybenzene			4.67	
Agarospirol 0.04 11.02			0.04	
Tetradecanoic acid			2.25	
5,5-dimethyl-2,2′-bithienyl			0.73	
4,5-dimethoxyphthalide			1.27	
Desmethylnomifensine			1.97	
Allyl-3-phenyl-3-trimethylstannybutanoate			0.72	
Saturated fatty acid (SFA)	Thailand Nutmeg seed Extracted by accelerated solvent extraction	GC–flame ionization detection	85.59	Obranović, et al., (2020).
Monounsaturated fatty acid (MUFA)			13.12	
Polyunsaturated fatty acid (PUFA)			1.29	
Lauric acid C12:0			0	
Myristic acid C14:0			75.69	
Palmitic acid C16:0			7.89	
Palmitoleic acid C16:1			0.12	
Heptadecanoic acid C17:0			0.84	
Octadecanoic acid. C18:0			1	
Oleic acid C18:1n9			13	
Linoleic acid C18:2n6			1.15	
Linolenic acid C18:3n6			0.15	
Icosanoic acid C20:0			0.17	
α-Thujen	Brazilian nutmeg extracted by hydrodistillation	GC–MS	1.71	Cossetin et al., 2021
α-pinene			10.51	
β-pinene			26.0	
Sabinene			9.16	
Myrcene			1.46	
α-Phella			0.92	
δ-3-care			1.61	
α-Terpin			4.93	
o-Cymene			2.97	
Limonen			4.67	
β-Phell			3.83	
γ-Terpi			8.51	
Terpino			1.59	
Cis-β-T			0.24	
4-Isopr			0.25	
4-Terpi			0.67	
α-Terpi			0.76	
Safrole			0.72	
Methyle			0.38	
Myristicin			0.76	

**Fig. 3:**
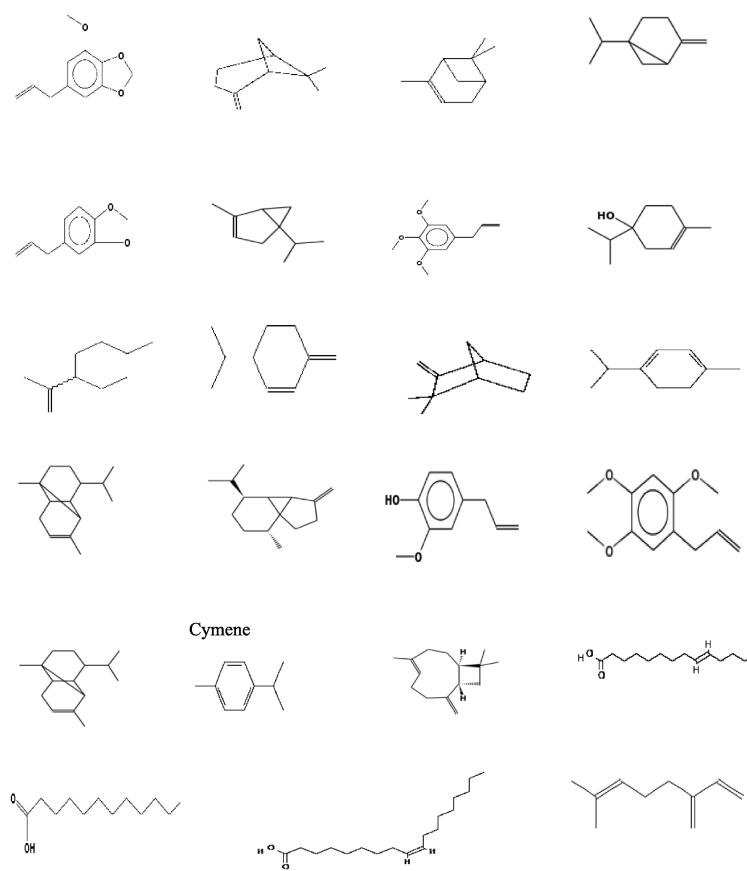
The molecular structures of some of the main active compounds of nutmeg extract adopted from the NIST library.

The reported chemical analysis of nutmeg oils and extracts confirmed that nutmeg is rich in phenolic compounds, terpenoids, flavonoids, and fatty acids (Spricigo et al., 1999). However, the concentration and presence of these groups varies with the geographical area of the seeds, freshness, types of extraction, and extraction conditions (Jaiswal, & Williams, 2017). Table 3 illustrates the chemical composition of nutmeg seed extracts from different geographical regions, which were extracted using different extraction methods. Machmudaha et al. (2006) confirmed similar properties of nutmeg supercritical extract to nutmeg essential oil because it contains most essential oil components such as terpenespolyphenol and limonene. On the other hand, from our reviewed data, it is clear that there is a substantial variation in the number of identified chemical compounds in nutmeg seed extract and their concentrations in different regions. Based on our review, we found that the number of identified chemical compounds in the nutmeg seeds ranged from 10 to over fifty compounds. Morsy (2016) identified 53 compounds present in nutmeg oil, with the presence of the main major group compounds such as myristicin, sabinene, elemicin, limonene, terpinen-4-ol, myristic acid α-pinene, and β-pinene. In addition, the concentrations of these identified compounds varied greatly depending on the extraction method or cultivation site. For example, myristicin concentration ranged from 2 % to 42 %, but it was also missing in some of the extracted nutmeg oil. In addition, other compounds were identified in minor concentrations using gas chromatography (GCTOFMS), which was not shown using other techniques, including anisole, camphor, cumene, copaene, cyclamen aldehyde, alfa-sarone, menthyl isovalerate, and menthone (Ibrahim & Al-Rawi, 1918). Fig. 3 shows the molecular structures of the main active compound of nutmeg extract, which were adopted from the NIST library.

### 3.8 Pharmacological and biological properties of nutmeg seed

Plants considered as potential natural products with significant pharmacological attributes, such as anti-inflammatory, antioxidant, antimicrobial, antidiabetic, and anticancer activities. It has a great role in treating and preventing various diseases due to its natural antioxidant, secondary metabolites, flavonoids, terpenes, alkaloid, phenol, and fatty acid. Therefore, potential attempts to treat various illnesses have raised a growing interest in combining pharmaceutical medications with natural therapies. In terms of both pharmacological and commercial activities, nutmeg seed has various pharmacological properties (Grover, et al., 2002). The extract of the nutmeg seeds is considered to be the fraction that contains the pharmacologically active components. Therefore, nutmeg deserves further research into its full benefits as a potential therapeutic and pharmaceutical agent. The following text describes the potential therapeutic properties of nutmeg seeds.

#### 3.8.1 Antimicrobial potential of nutmeg seed

Infections are health conditions that occur when microorganisms such as viruses, bacteria, fungi, or parasites invade our bodies and cause harm. Microorganisms such as bacteria are constantly evolving and developing new ways to resist antibiotics (Jalal Ahmed et al., 2023, Ahmed and Ganjo, 2019). Essential oils and other preparations from aromatic herbs have shown strong antibacterial effects against various fungi and bacteria (Hanif et al., 2010). In addition, many active compounds present in the nutmeg seeds, such as myristicin, carvacrol, −cymene, −pinene, −pinene, and −caryophyllene, have been proven to be potent antibacterial agents. The antifungal and antibacterial effects of nutmeg extract against Gram-positive and Gram-negative bacteria have been well demonstrated. Myristic acid and trimyristin were found to be the primary antibacterial components extracted from nutmeg seeds. Conversely, at a concentration of 12.5 µg/mL, the methanolic extracts of *Myristica fragrans* seeds inhibited the activity of Helicobacter pylori strains (Mahady et al. in 2005). These strains are known to be the main etiological factors associated with peptic ulcer disease, gastritis, primary gastric B-cell lymphoma, and gastric carcinoma. Therefore, nutmeg extract was found to be highly potent in treating gastrointestinal disorders and ulcers. According to Dorman and Deans (2000), the essential oil of *M. fragrans* exhibits significant inhibitory effects against a variety of bacteria, including bacteria that cause food poisoning, plant and animal pathogens, and spoilage. In a study conducted by Takikawa et al. (2002), the antibacterial properties of volatile oils extracted from nutmeg against non-pathogenic E. O157, and E. Coli were evaluated. The pathogenic strains of Escherichia coli O157 were significantly inhibited by the nutmeg extract, whereas the non-pathogenic strains were unaffected. In addition, all O157 strains were more sensitive to beta-pinene than non-pathogenic E. Compared with other Coli strains (Takikawa et al., in 2002). Table 4 displays the results of extensive research on the antimicrobial activity of nutmeg extract using various solvents and assays.

**Table 4:** The Antimicrobial Activities of Nutmeg Seed Extract using Different Solvents and Different Microbial Assays.

	Activity	Type of extract	Goal	Methods	Findings	References
	Anti-T. gondii & Cytotoxicity	Myrislignan	Study the mechanism of action	In vivo methods included CCK-8 assays, as for the *In Vitro qPCR* and staining were used.	In vitro, effects of myrislignan included the inhibition of *T. gondii* tachyzoite proliferation, as well as cell invasion by tachyzoites were reduced. Vero cells at concentrations less than 132 μg/ml showed no significant cytotoxicity effects	Zhang, Si, Li, Zhou, & Zhang, 2019
	Antibacterial activity	Nutmeg oil Components of the oil were (sabinene, myristicin, pinene and limonene)	The antibacterial activity of nutmeg was studied specifically in 2 regions: (Sulawesi and Central Java)	Water and stem distiller used for oil extraction. Antibacterial activity was carried out against pathogenic bacteria such as: *S.aureus, S. epidermis, S. Dysenteriae, S. Typhi.* In vitro disc diffusion method used for testing resistance patterns	The studied nutmeg’s oils were effective against: *S. aureus*, *S epidermis*, *S. dysenteriae* and *S. typhi*. Therefore, nutmeg oils can be considered as a potential natural antibacterial product.	Sarifah, Indira Lanti, & Dwi Pretti, 2017
	Antibacterial activity	Nutmeg seed methanolic Extract	Testing the extracted methanol from nutmeg and other organic origins effects on the growth of *S.mutans.*	Diffusion method using BHI agar later undergoing anaerobic incubation at 37 °C for 24 h.	Exhibited inhibition against growth of dental plaque forming bacteria.	Rosmalia & Marjoni, 2022
	Antimicrobial activity against oral pathogens	Nutmeg Oleoresin	Testing antimicrobial activity of silver nanoparticles using nutmeg oleoresin against *Streptococcus mutans* and *Enterococcus faecalis*	Agar well diffusion was used testing different concentrations of synthesized nutmeg oleoresin mediated silver nanoparticles.	AgNPs from Nutmeg Oleoresin has antimicrobial property, and the zone of inhibition increases as the concentration of AgNP's increases.	Pranati, Anitha, Rajeshkumar, & Lakshmi, 2019
	plaque control	Nutmeg oil	Comparing nutmeg mouthwash to 0.2 % CHX gluconate mouthwash according to the effectivity and plaque control	Two group was studied. Group A used 10 ml nutmeg mouthwash twice a day. Group B used 10 ml & 0.2 % CHX gluconate mouthwash twice a day, for 21 days.	Nutmeg mouthwash can be a good alternative to 0.2 % CHX gluconate mouthwash, both economically and the fact that it’s an organic compound.	Padol et al., 2022
	Antibacterial activity	Crude extract & Essential Oil	The goal was to inhibit the activity of efflux pump in MRSA.	• *Antimicrobial Susceptibility Testing, Using MIC & MBC* • *Polymerase Chain Reaction* • *Quantitative Real-Time PCR* • *Titration Assay* • *Agar Disc Diffusion*	Efflux pump inhibitors have potential as a new therapeutic agent such a nutmeg crude extract and essential oil as an alternative treatment. The synergistic effect between ciprofloxacin’s and Crude extract & Essential Oil revealed the most significant viability of MRSA.	Oo et al., 2021
	Antiviral activity	1. Malabaricones B and C Licarins A, B and C	*in sillico* evaluation of phytochemicals from nutmeg seed against COVID-19	• Molecular docking, • Testing solvent to extract compounds.	Compounds in the M. fragrans hold promise for future medical benefit against COVID19.	Ongtanasup et al., 2022
	Bacterial and fungal species	Essential oil	Assessing the antimicrobial activity against bacteria and fungi Gram-positive bacteria (*S.aureus*, *B.cereus*, *B. luteus*, *L.monocytogenes*) Gram-negative bacteria (*E.coli*, *K.pneumoniae*, *P.aeruginosa*, *P.vulgaris*) fungus (Candida albicans).	• Disc diffusion method	The highest sensitivity was exhibited by *B. luteus* however, gram negatives exhibited lower sensitivity.	Nikolic et al., 2021
	Antibacterial Activity	Essential oil	Determine the antimicrobial activity of hydrolats and essential oil by hydrodistillation in the presence and absence of magnesium aluminometasilicate as an excipient	• Serial dilutions in liquid medium • Cell Culture • ELISA to determine IL6 concentration.	A higher inhibition effect was exhibited by the oil and hydrolats with aluminometasilicate, EO inhibited *E. faecalis*, *S. mutans*, and *P. multocida*, entirely.	Matulyte et al., 2020
	Antimicrobial activity on refrigerated stored food	Nutmeg essential oil components: α-pinene, sabinene, β-pinene	To check the antimicrobial activity of (NEO) against: *E. coli*, *S.aureus*, psychrotrophic bacteria, and fungi.	Sage seed mucilage with Nutmeg essential oil used to coat food, then total viable count of pathogenic microorganisms was determined by diffusion agar assays	A significant synergistic effect was presented when combined with the antifungal agent nystatin.	Kiarsi, Hojjati, Behbahani, & Noshad, 2020
	Antimicrobial, antileishmanial, antilarvicidal potential.	aqueous extracts	The antimicrobial, anti-leishmanial, antidiabetic, antioxidant, and anti-larvicidal potential of the nanoparticles were tested.	ZnONPs and noncoated and ZnO-NP-coated antibiotics in different concentrations against UTI bacterial strains, (Agar Well Diffusion Assay for ZnO-NPs) and (Disc Diffusion Assay for Antibiotic Discs and Antibiotic-Coated ZnO-NPs)	• Antioxidant and antibacterial activities of the ZnO NPs were detected. • Results showed a significant effect of ZnO-NPs against larvae of Aegypti.	Faisal et al., 2021
	Antimicrobial activity	Oleoresin	nutmeg oleoresin was tested for its antimicrobial activity against bacterial pathogens that can be transmitted through contaminated food: *V. parahemolyticus*, *V. alginolyticus*, *L. monocytogenes*, *B. cereus*, and *E. coli.*	Antimicrobial activity was tested against some bacterial strains using the agar plate well diffusion method.	The mixture of nutmeg encapsulated with other substances such as: gum Arabic and sorghum starch showed inhibitory effects against *E. coli* and *B.cereus* but not on *V. parahemolyticus*, *V. alginolyticus*, and *L.monocytogenes.*	Arshad, Ali, Abbas, & Hasnain, 2018
	Antimicrobial activity against food spoilage	Essential oil	test nutmeg essential oil composition against pathogenic and food spoilage microbes: *S.aureus*, Shigella spp, *C.albicans*, and *A.niger.*	Disc diffusion method	Mixture of nutmeg oil, citronella oil, and patchouli oil inhibited the growth of C.albicans, A.niger and S.aureus.	Aisyah, Yunita, & Amanda, 2021
	Antimicrobial	Phytochemical constituents using methanol and acetone solvents	To examine the activity of this plant against four bacterial species: (*S.aureus* and *S.epidermidis*) (*E.coli*, and Klebsiella sp.), as well as one yeast (*C.albicans*)	Agar well diffusion	• Nutmeg acetone extract showed significant effect against microorganisms especially with towards *C.albicans* • The methanolic extract showed no inhibition zone.	Orabi et al., 2022
	Antibacterial and antifungal activity	Essential oil	Leaves of nutmeg were tested to discover its antibacterial and antifungal activities against: (*S.enterica*, *L.monocytogenes*, *S.dysenteriae*, *E.coli*, *P.aeruginosa*, A.niger and *F.oxysporum*)	Agar disc diffusion, PDA plates with EOs impregnated discs	Nutmeg leaf EO showed the highest inhibition activity against *S.dysenteriae* and, then against *L. monocytogenes*.	Fernando and Senevirathne, 2021
	Antibacterial activity	Aqueous extract	*E.coli*, *S.aureus*, Bacillus species and Streptococcus Spp.	Agar well diffusion	Higher antimicrobial effect was observed using hot water extract of nutmeg seed.	Sylvester, 2018
	Antimicrobial activity	Essential oil	Inhibitory activity against bacterial strains.	Antimicrobial activity test was applied according to broth microdilution test/ minimum inhibitory concentration (MIC) method.	Antimicrobial activity against almost all microorganisms were observed using the mixture of Nutmeg and cardamom essential oil.	ÖZKAN et al., 2018
	Antimicrobial activity of herbal extracts in root canal sealers.	Methanolic extract	A mixture of a*mla*, nutmeg and miswak was prepared and tested for their antimicrobial activities.	Agar diffusion test	A significant zone and largest zone of inhibition was observed when Endomethasone mixed with Nutmeg.	Devi et al., 2019
	Antimicrobial activity for preserving the quality bread	Oleoresin	The study used microcrystalline cellulose incorporated in the packaging material of gelatin base using oleoresins form natural sources: cloves, nutmeg, and black pepper.	Highest MIC of oleoresins was observed against *S. aureus* and *E. coli.*	Inhibition activity of nutmeg against *S. aureus* at 0.5 % and at 1 % against *Escherichia coli* was observed.	Figueroa-Lopez, Andrade-Mahecha, & Torres-Vargas, 2018
	Antibacterial Activities of Nutmeg	Essential oil	To test antimicrobial activity against gram positive and gram-negative bacteria.	Testing the minimum inhibitory concentration, maximum bactericidal concentration, using microdilution method	EO showed inhibition of bacteria at MIC ranging from 0.313 % to 10 %.	Wibowo, Febriana, Riasari, & Auilifa, 2018
	Nano emulsion of chitosan/nutmeg seed oil against microbial growth on strawberry	Essential oil	Evaluate (nano emulsion from chitosan/nutmeg seed oil) coating on fresh strawberry	Two suspensions were formed: (UTR-Emulsion and HPH-Emulsion), made as an edible coating, strawberries were coated in these suspensions to test activity against bacteria, mold and yeast	Best result in testing antimicrobial activity of EO was exhibited by high-pressure homogenizer-emulsion which was used for the coated strawberry.	Horison, Sulaiman, Alfredo, & Wardana, 2019
	Antimicrobial activity	Nutmeg seed oil mediated AgNPs	Evaluate the antimicrobial property of synthesized AgNPs of Nutmeg oil, against pathogenic strains.	Agar Well diffusion	*Nutmeg seed* oil showed effective antimicrobial activity, with least inhibition was shown against S.typhii. All other bacteria and fungi were observed to be sensitive to the *nutmeg seed* synthesized AgNPs.	PAULINE, Sangeetha, Manikandan, Loganathan, & Kalaiarasi, 2019
	Antifungal and antimicrobial activity	Aqueous extract of nutmeg	To test antimicrobial activity of Nutmeg seed extract-Graphene quantum dots against *S.aureus,* *P.aeroginosa, S.mutans, Salmonella sp, E.coli* and *M.Trichophyton*.	Minimum inhibitory concentration	Nutmeg extracted GQD showed bactericidal activity against MRSA and *E. coli* compared with the conventional commercially available mycoplasma removal agents, GQD was able to show similar results with a small dose 10 µgml-1	Thileepan, Thevanesam, & Kathirgamanathar, 2017
	Antibacterial activity	aqueous seed extract of nutmeg	To test the antibacterial activity of biosynthesized silver nanoparticles of nutmeg against (MDR) Salmonella enterica serovar Typhi	Methods used in the study included MIC and well diffusion methods.	Significant antibacterial activity was exhibited against G + and G- bacteria.	Balakrishnan et al., 2017

#### 3.8.2 Anti-inflammatory properties of nutmeg seed

**Fig. 4:**
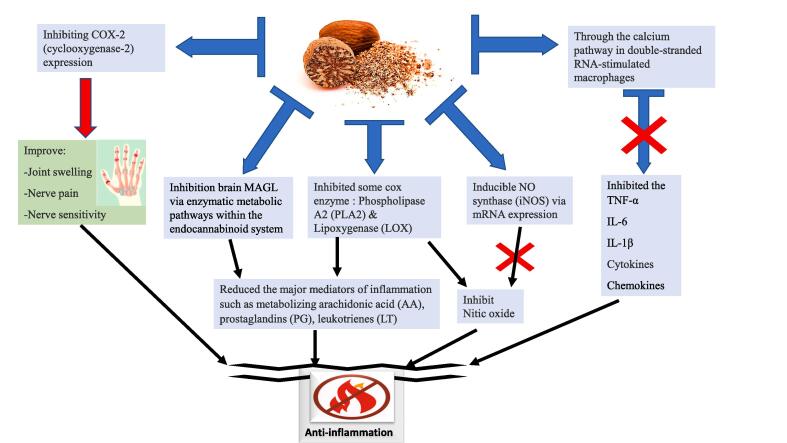
Anti-inflammatory mechanism of action of nutmeg. Nutmeg inhibited chemokines, nitic oxide, cytokines, and growth factors through the calcium pathway in double-stranded RNA-stimulated macrophages. It improved the joint swelling, nerve pain, and sensitivity by inhibiting COX-2 expression. Nutmeg inhibited the production of TNF-α, IL-6 and IL-1β, and NO via inhibition of inducible NO synthase (iNOS) mRNA expression. Nutmeg also inhibited some enzyme cox phospholipase A2 (PLA2), lipoxygenase (LOX), and nitric oxide synthase (NOS), which led to reduce the major mediators of inflammation such as metabolizing arachidonic acid (AA), prostaglandins (PG), leukotrienes (LT). COX-2: cyclooxygenase-2, TNF-α: Tumor Necrosis Factor Alpha, IL-1β: Interleukin-1 Beta, and IL-6: Interleukin-6, NO: nitric oxide, iNOS: inducible NO synthase, PLA2: phospholipase A2, LOX: lipoxygenase, AA: arachidonic acid, PG: prostaglandins, LT: leukotrienes.

Inflammation is the human body’s response to injury, infection, or any damaged tissue (Chen et al., 2018). It is a key contributor to several condition like cancer, autoimmune diseases, rheumatoid arthritis, cardiovascular diseases, blood pressure, hepatic injury, and obesity (Tsai et al., 2019, Wu et al., 2021, Ostro et al., 2014, Hage et al., 2017, Laird et al., 2013). Inflammation is characterized by pain, heat, swelling, redness, and tissue function disorder caused by the stimulation of numerous cytokines that cause inflammation, such as interleukin -6, IL-8, IL-1β, IL-1, and nitric oxide (NO) (Di et al., 2018, DiNatale et al., 2010, Manohar et al., 2018). Inflammation can be triggered by different signaling pathways, such as MAPK, NF-κB, and the JAK-STAT (Torres et al., 2011, Li et al., 2022, Lee et al., 2020). Several plant extracts have been used extensively in treating inflammation, including nutmeg (Francis and Sankari Malaiappan, 2022, Dkhil et al., 2019). Table 5, displays the results of the reported anti-inflammatory studies of nutmeg seed extract. Lee, & Park, (2011) revealed that the anti-inflammatory attribute of nutmeg is due to myristicin’s ability to suppress chemokines, cytokines, nitric oxide, and growth factors via the calcium pathway in double-stranded RNA (dsRNA) of stimulated macrophages. Nutmeg oil also alleviated the joint swelling, nerve pain, and sensitivity to pain in rats. The mechanism of action was by inhibiting cyclooxygenase-2 (COX-2) expression, which is an inflammatory key marker (Zhang et al., 2016). An in-silico study confirmed that myristicin blocked VEGFA, COX-1, EGF and HIF enzymes, signifying potential binding interactions (Al-Rawi et al., 2023b). Moreover, ethanolic extract of nutmeg inhibited in a dose-dependent matter the releasing of TNF-α, nitric oxide, IL-6 and IL-1β (Cao, et al., 2013; Dewi et al., 2015). Tumor necrosis factor α (TNFα) is a mediator of inflammatory and autoimmune functions and its excessive signaling activation can cause chronic pathological diseases (Jang, et al., 2021). Due to this, nutmeg extract has been frequently employed in traditional medicine as a topical application treatment to alleviate muscles, joints, and nerves pain (Al-Rawi et al., 2013). Nutmeg anti-inflammatory properties have been attributed to the presence of myristicin, a compound found in nutmeg, which exhibited anti-inflammatory properties (Liu et al., 2022). In addition, eugenol, another active compound present in nutmeg seeds, showed anti-inflammatory activity (Buckle, 2014). Thus, in the healthcare and medical industries, eugenol has been used in dental creams and analgesic ointment as well as in relieving sprains and rheumatism pain (Al-Rawi et al., 2011). This makes nutmeg a potent alternative to be used in the pharmacological industry. In addition, myrisfrageal A and B, dehydrodiisoeugenol (isolated compounds from nutmeg) inhibited the overproduction of nitric oxide (an essential marker of inflammation), with an IC50 of 18.5 & 21.2 μM respectively, via suppression of iNOS mRNA expression (Cao et al., 2015). In addition, flavonoids (presence in nutmeg) inhibited some enzyme that cause inflammation, such as nitric oxide, NOS, COX, phospholipase A2, and lipoxygenase. The inhibition reduced the major inflammation mediators such as metabolizing prostaglandins, arachidonic acid, leukotrienes and NO (Akinwunmi, & Oyedapo, 2014). Macelignan has also been reported as a potential health-promoting factor through its ability to inhibit inflammation and display strong antioxidant properties (Lee et al., 2012). Fig. 4 illustrates a summary of the nutmeg anti-inflammatory mechanism of action. These findings confirm the role and effectiveness of the anti-inflammatory and the chemo-preventive properties of nutmeg. This natural antidot has been extensively studied for its therapeutic effects, demonstrating promising results in reducing pain and inflammation. Its efficacy in pain management has made it a popular choice among health care professionals and patients alike. Thus, the use of nutmeg seed extracts in treating inflammatory related diseases warrants further investigation, especially in terms of its effectiveness and safety as a natural remedy.

**Table 5:** Illustrate the Application of Nutmeg in the Treatment of Chronic Incurable Diseases.

	Activity	Type of extract	Part of the plant	Goal	Methods	Findings	References
1	Anti-Inflammatory Activity	aqueous	seed	Nutmeg oil Effect on pain and inflammation of joints	In vivo	Nutmeg oil may alleviate joint swelling, allodynia, and hyperalgesia in rats by lowering COX-2 expression and substance P levels, suggesting its potential as a chronic pain treatment.	Zhang, et al., 2016
2		Extracts of uncooked, cooked, cooked and digested, in vitro nutmeg	Nutmeg seeds	To investigate how cooking and in vitro digestion affect nutmeg anti-inflammatory action.	Anti-inflammatory activity was assessed using the COX inhibition Cayman screening kit	Uncooked nutmeg exhibits anti-inflammatory effects by inhibiting COX-2 activity.	Baker, Chohan, & Opara, 2013
3		Water extract	The dried seeds of Nutmeg	Anti-inflammatory and antimicrobial activities of hydrolats and essential oil were assessed via hydrodistillation, with and without magnesium aluminometasilicate as an excipient.	anti-inflammatory activity was evaluated via: Cell Culture assay ELISA assay was used to determine IL6 concentration.	Oil and hydrolats with aluminometasilicate were more effective in inhibiting IL-6 in the presence of Poly I: C. The presence of magnesium aluminometasilicate as an excipient may alter and enhance the inhibitory effects of nutmeg essential oil and hydrolats.	Matulyte et al., 2020
4		nutmeg methanol and acetone solvents	(Myristica fragrans) seeds	To examine the impact of nutmeg acetone extract on COX-2 enzyme activity	Anti-inflammatory activities using Cayman COX inhibition kit	COX-2 activity was inhibited by nutmeg extract more than Aspirin (antiinflammatory drug)	Orabi et al., 2022
5		Ethanol extract of nutmeg	seed	To evaluate anti-inflammatory of nutmeg gel using in-vivo	Four formulas of gel were prepared with different concentrations. 2, 4, 8 %, and 12 %. The formulated gel was used to treat leg edema in male rat by carrageenan-induced paw edema method.	The formulation showed lower edema volume than the control. The highest anti-inflammatory was using 12 % concentration. At 8 and 12 % the anti-inflammatory activity was similar to the positive control.	Azis Ikhsanudin & Rais, 2021
6	CVD	Aqueous	seed	Change heart activity	In vivo (toad heart)	Increased ventricular contraction amplitude, sped up atrioventricular conduction, and induced sinus tachycardia. It also prolonged ventricular action potential duration and led to sinus bradycardia.	Saleh et al., 1989
7		Aqueous	seed	Cardiac remodeling	In vivo (MI) rats	Reduces cardiac remodeling by suppressing HIF-1 expression in mouse heart cells post-heart attack.	Liu et al., 2022
8		Aqueous	Seed	protection of CHD and mechanism of action	Rat Model of Myocardial Infarction	Protects heart tissue from heart attacks by reducing inflammation, oxidative stress, and cell death to prevent ischemia.	Lu, et al., 2018
9		Aqueous	seed	Cardiac fibrosis following MI	In vivo (MI) rats	Reduced cardiac fibrosis post-MI by regulating plasma metabolites to inhibit ECM-receptor interaction and TGF-1/Smad2 activation.	Yan, et al., 2022
10	hyperlipidemia	ethanolic	seed	Decrease lipid level in the blood	In vivo (oral administration in Albino rabbits)	Low-density lipoprotein and total cholesterol levels are reduced.	Ram, et al., 1996
11		aqueous	seed	Decrease the cholesterol and modulate lipid peroxidation	In vivo / hypercholesterolemic rats	Lowered cholesterol and LDL levels, decreasing lipid peroxidation and serum aminotransferase activities. Enhanced hepatic and cardiac antioxidant levels.	Onyenibe, et al., 2015
12	Hypercholesterolemia and atherosclerosis	Ethanol	seed	Decrease lipid level in the blood	In vivo (oral administration in Albino rabbits)	Prevents cholesterol buildup, removes aortic plaque, and boosts fecal excretion of lipids in rabbits fed seed extract.	Sharma, et al., 1995
13	Heat-stress tolerance	Oil	seed	Alleviate heat-stress in chicken.	In vivo (Korean native chicken)	Nutmeg extract boosts heat stress recovery in chickens by inhibiting lipid oxidation.	Hartanto et al., 2019
14	Diabetes	aqueous	seed	Using nutmeg in combination with glimepiride as alternative therapy for DMII	In vivo and in silico (in Swiss albino mice)	glimepiride and nutmeg promptly lowered blood sugar more than glimepiride alone.	Nasreen, et al., 2020
15	Diabetes	–	seed	Using nutmeg to increase glucose uptake by the muscle	In vitro (myotubes) and in vivo (mice suppressed post-prandial hyperglycemia)	Promote uptake of glucose in muscle to avoid post-prandial high blood glucose diabetes mellitus II.	Yoshioka, et al., 2022
16	Diabetes		seed	Effect of nutmeg extract on pancreatic tissue	In vivo (alloxan-induced diabetic rats)	100–200 mg/kg nutmeg extract lowered blood glucose, boosted insulin levels, and reduced oxidative stress in diabetic rats' pancreatic tissues.	Pashapoor et al., 2020
17	Diabetes	hydroethanolic	seed	Effect of silver nanoparticles from a hydroethanolic nutmeg extract on diabetes	in vitro	MFHENP inhibits alpha-amylase and alpha-glucosidase, delays glucose diffusion and uptake, similar to acarbose, promising for diabetes control.	Perumalsamy and Krishnadhas, 2022
18	Diabetes	dichloromethane-soluble extract	seed	Using Promalabaricone B in nutmeg extract to inhibit *α*-glucosidase enzyme.	In vitro (cell culture)	PMB induces hypoglycemic effects by upregulating AMPK and stimulating GLUT4 translocation, offering potential diabetes treatment.	Prabha, et al., 2021
19	Diabetes	menthol and methyl salicylate	seed	Effect of nutmeg extracts on pain in painful diabetic neuropathy patients.	In vivo (Painful diabetic neuropathy patients)	Both worst and average pain levels significantly dropped, along with reductions in pain's effects on walking, sleep, tingling, and mood.	Motilal and Maharaj, 2013
20	Diabetes	–	seed	Treat diabetes through reducing endoplasmic reticulum stress.	In vivo (obese diabetic mice) In vitro (cell culture)	Nutmeg activates PPAR-alpha/gamma, reduces ER stress, potentially treating type 2 diabetes. Macelignan in nutmeg enhances insulin sensitivity and corrects lipid metabolism.	Han et al., 2008
21	Obesity and diabetes	ethanolic	seed	Effect of *Myristica fragrance* extract on diabetes mellitus II and obesity.	In vitro (cell culture)	Nutmeg extract's AMPK compounds treat obesity, type-2 diabetes, and other metabolic disorders.	Nguyen et al., 2010
22	Obesity and diabetes	MeOH extract	dried seedl	Effect of *Myristica fragrance* extract on diabetes mellitus II and obesity.	In vitro (cell culture)	Meso-dihydroguaiaretic acid from *Myristica fragrans* stimulates insulin signaling by inhibiting PTP1B.	Yang, et al., 2006
23	Liver fibrosis	–	seed	effect of *myristica fragrance’s* methoxyeugenol in Cirrhosis	In vitro assay using human and murine cell line. In vivo CCl_4_ (carbon tetrachloride) −induced liver fibrosis in mice.	Methoxyeugenol could treat chronic liver disease and Cirrhosis.	de Souza et al., 2021
24	acute liver injury	CO2 supercritical extraction	seed	Protective effect of nutmeg extract on acute liver injury	In vivo (mice model). Thioacetamide (TAA)-induction of acute liver damage.	Nutmeg extract reduces TAA-induced liver injury by lessening oxidative stress and inflammation.	Yang et al., 2018
25	Hepatotoxicity	Aqueous	seed	High dose nutmeg administration affects oxidative stress, bile acid production and secretion	In vivo (male Kunmingmice)	Nutmeg causes liver injury through dose-dependent oxidative stress, elevating CYP450 levels, depleting antioxidants, and disrupting lipid metabolism.	Xia et al., 2021
26	hepatoprotective	Methanolic extract	Kernels	nutmeg seed Prevent Paracetamol-Induced Hepatotoxicity	In vivo (Rats)	Nutmeg extract has antioxidant, anti-inflammatory, and anti-apoptotic properties, possibly through activating the Nrf2/ARE pathway.	Dkhil et al., 2019
27	hepatotoxicity	Aqueous	seed	Hepato-protective and antioxidant	In-vivo (oral administration in rat)	Nutmeg extract showed antioxidant and hepatoprotective effects in isoproterenol-induced hepatotoxic rats.	Kareem, et al., 2013
28	Gastric Ulcer	–-	–	effects of nutmeg extract and Verapamil on gastric acid secretion	In vivo (Rabbit)	Reduced the volume, free and total acidity of gastric secretion	Jan, et al., 2005
29	Gastric Ulcer	ethanol	seed	protective effect of nutmeg extract on gastric ulcer	In vivo (Rats)	*Nutmeg* seeds treated vomiting, dyspepsia, and abdominal pain. It also protects against ethanol-induced ulcers.	Sattar et al., 2019
30	Cytotoxicity and oxidative stress	Aqueous methanol and ethyl acetate	seed	protective effect of macelignan against t-BHP-induced cytotoxicity in a HepG2	Invitro (human hepatoma cell line)	Macelignan inhibits cell growth and necrosis, reduces lipid peroxidation, and inhibits ROS production and DNA damage.	Sohn et al., 2007
31	Renal Ischemia	–	seed	Protect against renal ischemia reperfusion injury	In vivo / IRI rats	Macelignan in nutmeg protects against renal IRI by inhibiting inflammation, apoptosis, and boosting antioxidant defenses.	Long, et al., 2020
32	Renal	Raw	seed	Histological effect of nutmeg on kidney.	In vivo/Rats (oral administration)	High doses of oral nutmeg treatment in adult Wistar rats may adversely affect kidney function, potentially impairing excretory and metabolic activities.	Eweka and Eweka, 2010
33	Sexual function	ethanol	seed	Nutmeg and male sexual disorders	In vivo (rat)	Nutmeg's ethanolic extract boosts libido and potency, possibly by stimulating the nerves.	Tajuddin et al., 2005
34	Skin	Methanolic extraction	Seed	Protective photoaging effect of Nutmeg Macelignan from UV	Studying UV-irradiated human skin fibroblasts (Hs68) using RT-PCR, Western blot, DCFDA assay, and ELISA.	Regulates matrix metalloproteinases, key for skin aging, and modulates transforming growth factor-β (TGF-β), governing various cellular processes.	Lee et al., 2012
35		Methanolic extraction	Seed	Protective effects of isolated macelignan from Myristica fragrans HOUTT	In human keratinocytes (HaCaT), PAR-2 expression was investigate via RT-PCR, Western blot, and immunocytochemistry.	Macelignan decreased HaCaT PAR-2 mRNA and protein levels, suggesting its potential as a natural depigmenting agent to alleviate hyperpigmentation.	Hwang, 2010, Choi et al., 2011

#### 3.8.3 Cardioprotective of nutmeg seed

Chronic diseases, including heart disease, hypertension, chronic renal disease, arthritis, cancer, and diabetes, are the leading causes of disability and death in many countries. Chronic diseases are responsible for seven out of every 10 deaths around the world (Raghupathi & Raghupathi, 2018). Long-term illnesses known as chronic diseases are usually manageable but are not curable (Allegrante et al., 2019). Natural products, and functional foods offer natural remedies that have been used to treat many illnesses (Zhu et al., 2022, Kim et al., 2018). They play a significant role in regulating fundamental pathophysiological reactions such as oxidative damage, inflammatory processes, fibrosis, and hypoxia (Islam, 2022). Nutmeg seed poses a remarkable chemical composition that can prevent heart disease, diabetes, hyperlipidemia, and reduce lipid oxidation (Pratiwi et al., 2018, Parvin et al., 2023). The following texts explain in detail the potential effectiveness of nutmeg seed in treating some of the chronic diseases. Nutmeg seed has been vastly used in traditional medicine as a natural antidot for various health conditions, including cardiac diseases. Recent studies have shown that nutmeg seeds extract contains potent bioactive compounds that possess significant cardioprotective properties, making it a prosperous emerging prospect for the treatment of heart diseases (Yang et al., 2022, Pashapoor et al., 2020, Sharma et al., 1995). These properties are believed to be due to the various bioactive compounds in the extract, such as myristicin, safrole, and eugenol. Myristicin, the main active compound found in nutmeg, has the capacity to combat hyperlipidemia, neural damage, hyperglycemia, heart tissue damage, and hepatotoxicity due to it anti-inflammatory properties and its antioxidant activity (Liu et al., 2022). It has also been associated with improved heart metabolism that are alleviative for cardiovascular diseases (Liu et al., 2022). Apart from that, flavonoids, alkaloids, and other phytochemicals in nutmeg possess various biochemical and antioxidant effects associated with chronic health conditions including cardiovascular and Alzheimer's disease (Zhang et al., 2015, Deng et al., 2022). Quercetin a flavonoid found in nutmeg is responsible for improving cardiac metabolism through it antiplatelet, mitigating inflammation, estrogenic, antimicrobial, antiviral, antioxidation, antimutagenic properties (Roy et al., 2022, Salehi et al., 2020). The Table 5 illustrates the uses of nutmeg seeds in treating some incurable diseases including heart diseases. In conclusion, the use of nutmeg seeds extract as a natural remedy for heart diseases shows great potential. Nonetheless, the available evidence suggests that nutmeg seeds extract is a promising natural alternative to traditional medications for heart disease treatment. Additional investigation is essential to establish the optimal dosage, safety, and efficacy of nutmeg seeds extract as a treatment for heart diseases.

#### 3.8.4 Antidiabetic aspects of nutmeg seed

**Fig. 5:**
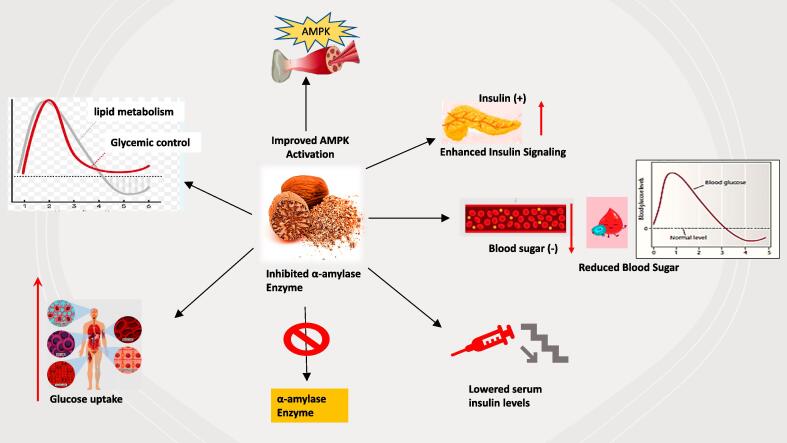
Anti-Diabetic Efficacy of nutmeg seed. Nutmeg stimulate insulin signaling and glucose reuptake by body cells, reduce blood sugar, ameliorate hyperglycemia and abnormal lipid metabolism. In addition, activated AMP- protein kinase (AMPK) enzyme in differentiated skeletal muscle cells C2C12, inhibited α-amylase and decreased serum insulin levels.

Diabetes represents a widespread chronic condition that affects millions of people globally. Diabetes is hallmarked by inadequate control of blood glucose level due to insulin deficiency and/or insulin insensitivity (CDC, 2022). Although there are several synthetic drugs available for diabetics, however, they often come with undesirable side effects. Therefore, natural products have been explored as an alternative treatment option for diabetes. In traditional medicine, plants, herbs, and spices have shown promise as treatment options for diabetes, its complications, and its management. Nutmeg extract was reported to stimulate insulin signaling and glucose reuptake by body cells and reduce blood sugar (Broadhurst et al., 2000). Nutmeg extract exhibited antidiabetic and β-cell protection attribute due to its corrective ability to enhance lipid metabolism and hyperglycemia (Arulmozhi et al., 2007). Nutmeg seeds extract also stimulated the AMP-activated protein kinase enzyme in differentiated skeletal muscle cells C2C12, which makes nutmeg a good candidate in treating obesity, diabetes, and other metabolic disorders (Nguyen, et al., 2010). Another standpoint, the inhibition of α-amylase plays a crucial role in the management and treating of diabetes disease. The inhibition of α-amylase slows down the conversion of starch to glucose which lead to the glucose levels reduction. As a result, anti-diabetic drugs are crafted and developed using α-amylase inhibitors. The water extract of nutmeg seeds inhibited the α-amylase activity with 28.96 % using 1 mg/ml (Bhutkar et al., 2018). On top of that, benzene extracts of nutmeg showed higher anti-amylase activity (57.80 %) with IC50 of 2.25 ± 0.28 mg/ml while methanol extract of nutmeg showed less inhibition with (16.20 %) using 2.5 mg/ml (Hemlata et al., 2019). Nutmeg seed is remarkably valuable in diabetes treatment of hyperinsulinemia type due to its capacity in reducing the serum insulin levels (Pereira et al., 2019). In conclusion, nutmeg seeds can increase insulin sensitivity, improve glucose metabolism, and attenuate the risk of diabetic-related complications as shown in Fig. 5. Therefore, nutmeg may be applied in formulating anti-diabetic drugs and in controlling or to manage the complications of diabetes. The uses of nutmeg seeds in treating diabetes disease are illustrated in Table 5. However, further research is still needed to ascertain the safety and efficacy of nutmeg seeds in the treatment of diabetes.

#### 3.8.5 Apoptotic and anti-cancer of nutmeg

**Fig. 6:**
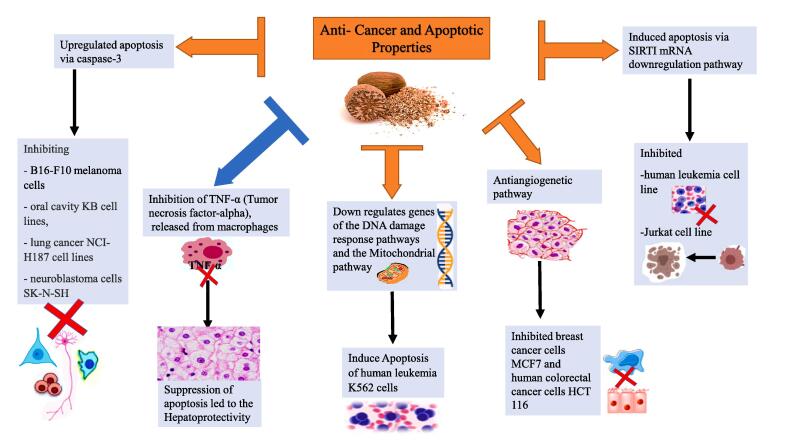
Anticancer Mechanism of nutmeg seed through Apoptotic Pathways. Nutmeg inhibited melanoma cells B16-F10, oral cavity KB cell lines, and lung cancer NCI-H187 cell lines, and apoptosis via caspase-3. Induced apoptosis in Jurkat cell proliferation significantly and human leukemia cell line through Sirtuin-1 (SIRTI) mRNA downregulation pathway. Nutmeg inhibited breast cancer MCF7 cell lines and human colorectal cancer cells HCT 116 through angiogenesis pathway. Nutmeg inhibited leukemia K562 through DNA damage response pathways and the mitochondrial pathway. It also induced hepato-protectivity, through inhibiting Tumor necrosis factor-alpha (TNF- α) which is released from macrophages that lead to the suppression of apoptosis. Nutmeg induced cytotoxicity against human neuroblastoma cells SK-N-SH by apoptotic mechanism.

Cancer is one of the main or the primary contributors to global mortality (Lim, 2002). Breast cancer was ranked globally as the primary cause of female cancer-related death, constituting approximately 25 % of all diagnosed cancer cases (Lei, et al., 2021). Epidemiological studies indicate variations in cancer prevalence across different cultures. Interestingly, countries with low cancer incidence often demonstrate a correlation with elevated spice consumption, suggesting it as a contributing factor. Besides genetics, cancer has been linked to lifestyles and diets, with a ratio of (95–99 %) and (5–10 %) respectively (Anand, 2008, Monahan et al., 2020). Moreover, migration from one’s native country to an adopted one undoubtedly amplifies the risk and incidence of an individual developing the same cancer as the rest of the population in the adopted country (Al-Rawi et al., 2011). Thus, plants, herbs and seed extracts have gained tremendous attention in recent years because they are found to be potent in preventing or treating cancer with a good pharmacological safety profile (Hanif et al., 2023; Al-Rawi et al., 2011, Ibrahim et al., 2011). The extraction method also proved to have a promising improvement in yield activity (Ab Rahman et al., 2011). Nutmeg essential oils have therapeutic and protective properties against carcinogenesis. Nutmeg seed extract inhibited B16-F10 melanoma cells with IC50 21.83 µg/mL for ethanol extract, ethyl acetate (21.66 µg/mL), and n-hexane (47.53 µg/mL) by inducing apoptosis via caspase-3 (Susianti et al., 2021). In recent research, the use of compounds isolated from nutmeg seeds on oral cavity KB cancer cell lines, and lung NCI-H187 cancer cell lines showed significant in vitro cytotoxic activity with IC50 values of 5.9 and 6.3 μM, respectively (Chumkaew, & Srisawat, 2019). Nutmeg methanolic extract also significantly inhibited the proliferation of immortalized Jurkat cell and prompted apoptosis of human leukemia cancer cell lines at concentrations of 50 and 100 μg/mL (Chirathaworn et al., 2007). The functional mechanisms behind this activity involves the Sirtuin 1 (SIRTI) mRNA downregulation pathway, which is triggered by the polyphenols in nutmeg. Polyphenols were found to stimulate cells proliferation and the initiation of SIRT1 gene expression mechanisms (Ibrahim et al., 2017). The extraction technique of supercritical nutmeg seed extract has been shown to influence the extract properties. The supercritical nutmeg extract has mild activity against breast cancer cells MCF7 and the human colorectal HCT 116 cancer cell line (Al-Rawi et al., 2011, Al-Rawi et al., 2023c). Nutmeg extract also significantly inhibited the angiogenesis (blood vessels) formation in an *ex vivo* 3D rat model using 100 µg/mL (Al-Rawi et al., 2023a). This inhibiting of blood vessel growth was behind the growth inhibition of breast cancer cells (Al-Rawi et al. 2023). Anti-angiogenic therapy stands out as a highly promising approach in treating and controlling cancer and angiogenesis-dependent diseases, including rheumatoid arthritis, diabetic retinopathy, obesity, cardiovascular disease, and lymphopenia (Al-Rawi, Ibrahim & Ahmed, 2023). The anti-angiogenic property of nutmeg seeds was attributed to the composition of the nutmeg seed extract and the presence of some active compounds such as aromatic ether group, terpenes, flavonoids, and phenolic compounds (Al-Rawi, et al., 2011). These bioactive compounds exhibit anticancer and anti-inflammatory properties (Crozier et al., 2006). On top of that, the presence of benzodioxoles composites in nutmeg, such as myristicin, which has antitumor and antioxidant activity (Gupta et al., 2016). Additionally, wide varieties of bioactive compounds are present in many seeds and spices including nutmeg. Sesquiterpenes (one of the nutmeg components) showed remarkable pharmacological activities against cancer (Modzelewska et al., 2005). Ahmad et al., (1997) reported the inhibitory effect of myristicin on lung tumor formation in mice, providing evidence that myristicin functions as a preventive mediator. Myristicin from nutmeg also induced apoptosis in human leukemia K562 cells via downregulating the activity of genes associated with DNA damage response and the mitochondria pathways (Martins, et al., 2014). In the same manner, nutmeg seed extract exhibited a potent hepatoprotective activity (Morita et al., 2003). This action was also linked to myristicin, which is the primary active component of nutmeg. The functional mechanism through which myristicin provides hepatoprotective effects includes restraining TNF-α, which is released from macrophages which lead to the suppression of apoptosis. Apart from that, myristicin induced a toxic impact against SK-N-SH (human neuroblastoma cells) via apoptotic pathways (Lee et al., 2005*)*. Likewise, limonene one of the main compounds in nutmeg, also has anticancer properties that add extra power to the nutmeg effect. In addition, nutmeg also contains phytosterols. Phytosterols are linked to various functional processes mechanisms, including angiogenesis, cancer-cell inhibition, and the promotion of apoptosis in cancer cells (Dahham, et al., 2018). Fig. 6 shows the anticancer mechanism pathways of nutmeg seeds. In summary, based on our review, nutmeg extract can be used as a treatment and as a chemopreventive agent for different types of cancer. Moreover, nutmeg seeds contain compounds that can be used either independently or as adjuvants to existing chemotherapeutic agents, aiming to boost their effectiveness while minimizing associated toxicity.

#### 3.8.6 Antioxidant properties of nutmeg seed

The popularity of natural antioxidants is on the rise globally as health-promoting bioactive compounds. They are often derived from many parts of the plants, such as vegetables, fruits, herbs, and spices (Anwar et al., 2018). Antioxidants are a broad group of compounds that include vitamins, minerals, phenols, and carotenoids. It displays a diverse range of therapeutic effects, such as antiaging, antimicrobial, antidegenerative disorders, antiinflammation, and anticancer effects (Adwas et al., 2019). Seed oils have been used for millennia as a treatment in traditional medicine (Liu, et al., 2022; Ahmad, 2009). Seed oils possess a rich composition of free carrier capacity and natural antioxidants. It exhibits diverse mechanisms such as neutralization free radicals, reducing lipoperoxidation, and enhancing our natural enzymatic defenses (Moussa et al., 2019). In addition, free radicals, and reactive oxygen species (ROS) have a pivotal impact on ion transportation, gene expression, cancer, and apoptosis (Azad and Iyer, 2014). They interact with a number of cellular molecules and metabolites, causing cellular damage and diseases (Asif, 2015). Antioxidants are associated with health improvement by constraining the production of free radicals and ROS (Noguchi and Niki, 2019, López-Pedrouso et al., 2022). The N-hexane extract of nutmeg seeds contains a substantial number of various antioxidants (Parle et al., 2004). Flavonoids and alkaloids have been identified as key antioxidants present in nutmeg (Spricigo et al., (1999). Flavonoids are antioxidants made of polyphenolic composites. It is well known for treating of cancer due to its capacity in blocking the growth of cancer (Davis, 2001). Murcia et al., assessed the antioxidant properties of nutmeg in 2004. Their comparison and evaluation included nutmeg, propyl gallate (E-310), BHA butylated hydroxyanisole (E-320) and BHT butylated hydroxytoluene (E-321). The nutmeg antioxidant capacity using the Trolox equivalent antioxidant capacity (TEAC) assay was higher than that of BHT. They suggested that nutmeg seeds exhibited the highest protective property using the deoxyribose assay. They also confirmed that nutmeg enhanced the stability and oxidation of oils at 110 °C, such as olive oil, corn oil, sunflower oil, margarine, and butter. In the same manner, the antioxidant and radical-scavenging activity of nutmeg using the DPPH radical assay has been reported among other oils by Tomaino et al., (2005). They demonstrated strong antioxidant and free radical-scavenging potential at room temperature using DPPH radical assay. The effectiveness varied among these oils, whereas nutmeg was stronger than basil, oregano, and thyme. Overall, the nutmeg antioxidants properties place it as a fit broadcast potential candidate and an agent to prevent and treat many health conditions. In Table 6, the antioxidant compounds present in nutmeg seed extract using different antioxidant methods are illustrated.

**Table 6:** The antioxidant activity of nutmeg seed extract.

Extract	Antioxidant method	Finding	Reference
crude nutmeg extract in methanol 2.4 μL/mL	The 2,2-diphenyl-1-picrylhydrazyl (DPPH) free radical scavenging assay and the β-carotene-linoleic acid assay.	DPPH assay result showed that eugenol followed by methoxyeugenol had higher activity than BHT. While isoeugenol had higher activity than α–tocopherol. β-carotene-linoleic acid assay, the showed α-tocopherol had higher antioxidant activities than BHT and isoeugenol, followed by methoxyeugenol and the weakest activity was eugenol.	Kim,et al., 2010
acetone, ethanol, methanol, butanol, and water extract of nutmeg	The DPPH (1,1-diphenyl-2-picrylhydrazyl) radical scavenging activity of the various extract (0.025–2 mg/mL) or BHT (0.025–1.0 mg/mL) was measured using the method of Brand-Williams, Cuvelier, and Berset. BHT was used as positive control.	The acetone extract showed 93.12 ± 1.48 mg gallic acid equivalents (GAE)/100 g dry weight total phenolic content, DPPH scavenging activity of 63.04 ± 1.56 %, chelating activity of 64.11 ± 2.21 % and 74.36 ± 1.94 % inhibition of β-carotene bleaching, at 1 mg/mL extract concentration	Gupta, Bansal, Babu, & Maithil, 2013
Nutmeg extracted by acetone	DPPH radical scavenging capacity	DPPH radical scavenging capacity of the acetone extract as well as its fractions was comparatively lower than that of green pepper phenolics.	Chatterjee et al., 2007
Essential oil and oleoresins (ethanol, ethyl acetate, and *iso*-propyl alcohol) of Myristica fragrans	scavenging effect on DPPH, reducing power, and chelating effect was determined.	The essential oil and ethanol oleoresin showed better activity compared to other tested oleoresins and synthetic antioxidants, butylated hydroxyl anisole and butylated hydroxyl toluene.	Kapoor et al., 2013
Six isolated compounds from nutmeg seed of Myristica fragrans licarin-B, dehydrodiisoeugenol malabaricone B, malabaricone C, β-sitosterol, and daucosterol.	Antioxidant activities of the isolated compounds were studied using oil stability index (OSI), reducing power, ABTS scavenging, and DPPH scavenging methods.	The results showed that Malabaricone C is an efficient antioxidant agent which exhibits a stronger antioxidant activity than the commonly used synthetic antioxidants in all studied methods	Hou, Wu, Wang, & Weng, 2012
essential oil from nutmeg seed.	The antioxidant activity was examined by DPPH assay using spectrophotometric.	The nutmeg essential oil showed a good antioxidant activity after incubation (EC50 = 1.35 ± 0.003 mg/ml)	Nikolic et al., 2021
Methanol extract potential of flesh, seed and mace of nutmeg (Myristica fragrans Houtt)	(DPPH), ferric-reducing antioxidant power (FRAP), ferrous ion chelating activity and antioxidant activity assay in a linoleic acid system with ferrothiocyanate reagent (FTC).	Flesh, seed, and mace extract well inhibit the linoleic peroxidation. Tannin, flavonoid and terpenoid were found in seed and mace extract, whereas flesh extract contains flavonoid and terpenoid.	Assa, Widjanarko, Kusnadi, & Berhimpon, 2014
Polyphenol extracts of nutmeg.	O brine-shrimp lethality assay, phytotoxicity test, DPPH, and superoxide anion radical scavenging as well as BSA-glucose antiglycation assay	Nutmeg extract exhibited a cytotoxic and phytotoxic potential with LD50 of 4359.70 and 1490 μg/mL respectively.	Kazeem, Akanji, Hafizur, & Choudhary, 2012
Methanol and acetone extract of nutmeg	Phenol content & radical scavenging activity were measured quantitatively using (DPPH) (µPADs) methods	The extract contains a high concentration of phenolic compounds (0.6217 mg/ml) and the DPPH assay for acetone extract indicated a high amount of antioxidant compounds.	(Orabi et al., 2022)

#### 3.8.7 Psychotropic action of nutmeg seed

**Fig. 7:**
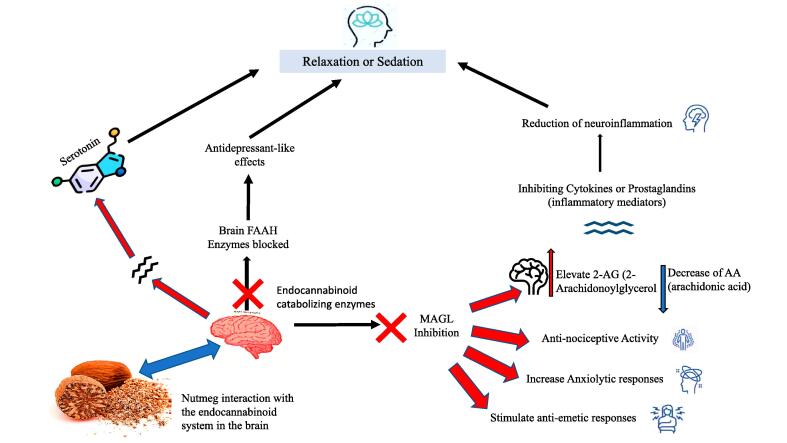
Psychotropic mechanisms of action and narcotic effects of nutmeg seed. Nutmeg stimulates the release of serotonin that creates a feeling of relaxation or sedation. This mechanism is triggered by the nutmeg interaction with the endocannabinoid system in the brain. Nutmeg extract inhibited the and monoacylglycerol lipase (MAGL) and the endocannabinoid catabolizing enzymes fatty acid amide hydrolase, The inhibition of MAGL stimulates the anti-nociceptive, anxiolytic, and anti-emetic responses, elevate brain levels precursor of inflammatory mediators, resulting in the reduction of neuroinflammation.

Nutmeg ingestion has been reported to cause symptoms similar to those of anticholinergic poisoning, including giddiness, tingling, euphoria, and hallucinations such as time–space distortion, detachment from reality, limb separation, and fear of death (Pawar, 2023, Desai, 2016). Nutmeg has a reputation for its sedative, hallucinogenic, and anticholinergic poisoning properties (Hausner, & Poppenga, 2012). However, to reach these psychogenic effects, about 1–3 seeds or 5 g up to 30 g of the ground nutmeg seeds is needed, as 7 g of ground nutmeg equals 1 tablespoon (Holstege, 2005). Recently, n-hexane extract of nutmeg has been found to be very safe with LD50 > 2000 mg/kg, while the nutmeg extract showed antidepressant effects over various nervous system components related to serotonin and norepinephrine. (Iwata et al., 2022). Nutmeg is also recognized for its antidepressant and anxiogenic properties. In a recent study, nutmeg extract showed a great effect on the nervous system, and could treat anxiety, behavioral agitation, and insomnia (El-Alfy, et al., 2019). This is due to the properties of nutmeg components, which can stimulate the serotonin secretion, that generates relaxation or sedation. This mechanism is triggered by the nutmeg interaction with the endogenous cannabinoid system in the brain. The endocannabinoid system (ECS) is responsible for regulating various physiological processes, incorporating immune function, appetite, mood, and sleep. Nutmeg extract inhibits monoacylglycerol lipase (MAGL) and the endogenous cannabinoid enzyme fatty acid amide hydrolase (FAAH) (El-Alfy, et al., 2016). The inhibition of MAGL has been shown to stimulate anti-emetic responses that are anxiolytic and anti-nociceptive (Mulvihill & Nomura, 2013). Furthermore, MAGL inhibition has a significant impact on the brain precursor levels of inflammatory, resulting in the reduction of neuroinflammation (Kasatkina et al., 2021). This is due to the elevation of 2-AG (2-arachidonoylglycerol) and a marked decrease in AA, which is a crucial building block of prostaglandins, a pro-inflammatory mediator (Deng, & Li, 2020). This will result in a significant decrease in neuroinflammation. In addition, the nutmeg n-hexane extract significantly enhanced the memory and learning activity of mice at 5 mg/kg body weight (Parle et al., 2004). Fig. 7 shows the psychoactive, psychostimulant, and mechanisms of action of nutmeg seed. The improving effect of nutmeg extract was attributed to its procholinergic activities and the existence of various antioxidant compounds individually or synergistically. Nutmeg seeds have been found to provide relief from chronic and persistent pain in different parts of the human body (Zhang et al., 2016). However, caution must be taken with nutmeg consumption for the long term as it could have adversative negative effects on the auditory responsiveness of humans (Adjene, & Nwose, 2010). In summary, the use of nutmeg in the development of medications is highly recommended. However, it is imperative to note that the psychoactive component of nutmeg may require modification or combination with other compounds to maximize therapeutic benefits while minimizing potential side effects and abuse potential. Therefore, further research is crucial to establish the ideal conditions for developing nutmeg-based medication.

### 3.9 Toxicological properties of nutmeg seed

Nutmeg seeds are famous for their bad reputation as being toxic and possessing narcotic properties (Weil, 1971). Few cases of nutmeg intoxication have been reported. A case of intoxication was reported of nutmeg seeds powered consumption by a 23-year-old college student (Abernethy and Becker, 1992). A 16-year-old student case of nutmeg poisoning was also reported after consuming the grinded raw seeds (McKenna et al., 2004). A case was reported of a disoriented young male (17-year-old) who was walking back and forth and having noticeable conversations with himself due to an overdose of nutmeg (Beckerman & Persaud, 2019). However, nutmeg ingestion alone is not likely to cause death, and symptoms vanish within 24 h (Smith, 2014). A quantity of 5–30 g of nutmeg powder is deemed toxic, yet not life-threatening or death occurred (Brenner et al.,1993). However, even with higher doses of nutmeg seeds ingestion (20–80 g of powder), no life-threatening condition was reported (Stein, Greyer, & Hentschel, 2001). However, these reports were for consuming raw nutmeg seeds but not extract or oil of nutmeg seeds. Many extraction methods have proved to be efficient in producing safe and toxic-free extracts (Zhang et al., 2019, Ibrahim et al., 2017). Supercritical extraction is one of the most efficient techniques for producing safe and clean products. Modifying the supercritical extraction parameters can produce different fractions with varied compositions and less toxicity (Ibrahim and Al-Rawi, 2018, Al-Rawi et al., 2011). In addition, supercritical nutmeg extract and its bioactive component myrislignan exhibited potent in vivo protective properties against thioacetamide-induced liver toxicity (Yang et al., 2018). Thus, the use of nutmeg extract can provide a potent ingredient in the pharmaceutical and food industries to provide a safe profile product that is toxin-free. More rigorous investigations are needed to establish the efficacy and safety of nutmeg seeds and to identify the optimal dosages and formulations for treatment.

### 3.10 The pharmacokinetic of nutmeg seed

The study of pharmacokinetics focuses on understanding how a particular substance is processed within the body after it has been administered. The onset, duration, and effectiveness of a drug are largely influenced by its pharmacokinetics, which helps professionals to adjust drug dosages precisely and efficiently, leading to personalized pharmacotherapy (Kimura et al., 2010). To comprehend the therapeutic properties of nutmeg seeds, it is crucial to understand its pharmacokinetic properties, which include the absorption, distribution, metabolism, and excretion in the body (ADME). However, our knowledge of the pharmacokinetics of nutmeg, especially at safe doses, is limited. The absorption of nutmeg seed and its active ingredients occurs after oral consumption in the gastrointestinal tract (Yakaiah et al., 2021). In the gut, the active compounds are released and absorbed by the intestine lining, where they pass to the bloodstream and circulates all over the body (Kazlauskaite et al., 2023). Nutmeg elements are quickly absorbed such as elemicin and myristicin, and their concentration are detected in the bloodstream within couple of hours after consumption. The absorption of myristicin is straightforward after oral consumption, processed extensively, and 73 % of the total dose has been excreted in the urine of experimental animals in the form of as CO2 within 24 hr (Al-Ajlouni et al., 2017: Beyer et al., 2006). In another study, 2 μg/ml of myristicin was detected in the blood after 8 h, when 14–21 g or 280–420 mg/kg of body weight of nutmeg powder was consumed, which is equal 2–3 tablespoons. However, many factors can affect this process such as, presence of enzymes, food, pH, medication and formulation. In a prominent death case, a 4 μg/ml of myristicin has been detected in the post mortal serum of a 55-year-old woman. However, it has been found that the cause of death was due to the toxic effect of flunitrazepam (0.072 μg/ml) with myristicin (Stein et al., 2001). In addition, certain factors might affect the absorption of nutmeg elements in the gastrointestinal environment including solubility, permeability and unstable, yet, certain compounds can pass through the intestine lining and enter the bloodstream (Kazlauskaite, et al., 2023). Moreover, myristicin and elemicin (compounds present in nutmeg), has the ability to cross the blood barrier of the brain by the P-glycoprotein through the passive diffusion transport mechanism (Wu,et al.,2016).

Nutmeg constituents and their metabolites are distributed via the bloodstream through all over the body using a distinctive distribution kinetics. This distribution mechanism of these compounds is affected by the fat solubility and their molecular weight (Onetto & Sharif, 2023; Blokhina, et al., 2021). In the body, following absorption, the drug will be processed in the body and broken down into several metabolites in a mechanism called drug metabolism. Several important biotransformations will be produced from nutmegs to form reactive metabolites via their metabolic processes.

Several nutmeg metabolites are known as valuable indicators of nutmeg metabolism, which are formed from nutmeg constituents including myristicin, safrole and elemicin. The liver serves an essential function in the initial metabolism of nutmeg, as majority of metabolic process are carried out by cytochrome enzymes (CYP450) (Zhao et al., 2009). It has been found that 4 g/kg of nutmeg stimulate the CYP450 enzymes levels (Xia et al., 2021). These enzymes are known to metabolize drug, and are mostly expressed in the liver (Zhao et al., 2021). In humans, almost 80 % of oxidative metabolism and 50 % of the overall excretion of common drugs are eliminated by these enzymes. CYPs enzymes has the ability to influence the drug's action, responses, drug resistance, bioavailability, and safety, through the act of metabolism (Zhao et al., 2021). Nutmeg major constituents myristicin and elemicin, endure a metabolic process called phase I, that forms diverse reactive metabolites with potential pharmacological property, such as tautomers and quinone (Yang et al., 2015). Moreover, several pathways of myristicin metabolism have been suggested, including hydroxylation, demethylenation, amination of the allyl group, and O-demethyl(en)ation being the primary biotransformation process of alkenylbenzenes. There are several metabolic pathways of nutmeg ingredients. These include sulfate conjugation as the primary phase II metabolic pathway, various combinations of dealkylation and hydroxylation for each nutmeg ingredient, formation of phase I metabolites through processes like hydroxylation and dealkylation, formation of metabolites phase II after sulfate conjugation, leading to the detection of multiple metabolites, and specific transformations such as demethylation, methylation, hydroxylation, oxidation, and hydrolysis of nutmeg ingredients, resulting in the generation of distinct metabolites (Neukamm et al., 2022).

Several metabolites have been identified in animal urine via oral route studies. These include 1-(3′,5′-dimethoxy-4′-hydroxyphenyl)-prop-2-ene, 1-hydroxy-1-(3′,4′-methylenedioxy-5′-methoxyphenyl)-prop-2-ene,1-(3′,4′-dihydroxy-5′-methoxyphenyl)-prop-2-ene, 2,3-dihydroxy-1-(3′,4′-methylenedioxy-5′-methoxyphenyl)-propane, and 1-(3′,4′-methylenedioxy-5′-hydroxyphenyl)-prop-2-ene (Beyer et al., 2006). On the other hand, the last three aforementioned metabolites were detected in the urine of rats and guinea pigs by Oswald et al. (1971), using intraperitoneal injection of myristicin, and found that these were the respective major urinary metabolites. Based on these findings, it has been suggested that the frequency of numerous pathways of myristicin metabolism might fluctuate with relation to species and administration route. Moreover, these metabolic pathways may behind the potential pharmacological property observed from nutmeg seed extract. Metabolites of myristicin, safrole and elemicin (Nutmeg components) have been detected in human urine as well, in addition to animals. Several metabolites have been detected in the urine of a patient who had an overdose of 5 nutmeg seeds (Beyer et al., 2006). These detected metabolites were: 1-(3′,5′-dimethoxy-4′-hydroxyphenyl)-prop-2-ene, and 2,3-dihydroxy-1-(3′,4′-methylenedioxy-5′-methoxyphenyl)-propane, that have been obtained from myristicin, whereas 1-(3′,4′-dihydroxy-5′-methoxyphenyl)-prop-2-ene, and 1-(3′,4′-methylenedioxy-5′-hydroxyphenyl)-prop-2-ene, have been obtained from a combination of nutmeg constituents. The 5-allyl-1-methoxy-2,3-dihydroxy-benzene is myristicin major metabolites, which is formed by myristicin metabolism by the liver microsomes. This biotransformation is accelerated by the CYP1A2 enzyme which is one of the CYP3A4 enzymes (Yun, et al., 2003). Myristicin could also somewhat metabolized into N-acetylcysteine, and 3,4-methylenedioxy-5-methoxyamphetamine (MMDA) which has psychedelic properties, while elemicin is metabolized partly to 3,4,5-trimethoxyamphetamine (TMA), both of which are amphetamine derivatives ((Zhu et al., 2019, Casale et al., 2023).

Generally, the three main metabolites typically detected after nutmeg intake are safrole, myristicin, and elemicin. Approximately 25 metabolites have been spotted in human blood and urine samples, which were formed in the body from metabolizing nutmeg seed constituents, safrole elemicin, and myristicin, via their metabolic pathways and their following biotransformations (Manier et al., 2021). In another study, 8 different metabolites have been detected when consuming 1.5 g of nutmeg within 18 h (Usui et al., 2023). These finding give a glimpse about the elimination and excretion of nutmeg ingredients. The metabolites of nutmeg seed could be mainly excreted by the biliary and renal excretion (Manier et al., 2021, Usui et al., 2023). It has been suggested that nutmeg metabolites are excreted in their particular coupled forms (Lee et al., 1998). The half-life elimination of nutmeg compounds is affected by exposure route, metabolism and dose (Neukamm et al., 2020). Additionally, the presence of some nutmeg metabolites in the body might be prolonged by enterohepatic circulation (Yang et al., 2015, Song et al., 2019). In summary, nutmeg seed possess exceptional pharmacokinetic characteristics, comprising their absorption in the alimentary tract, distribution throughout the blood stream, metabolized in the liver, and excreted through biliary and renal elimination pathway. Up to date, there have been no FDA regulations regarding the allowed dose of nutmeg, myristicin, as a powders, oil, or oleoresins in food or pharmaceutical industries. Understanding the pharmacokinetic effects of nutmeg is crucial in nutmeg-based treatments to optimize their dose treatments, interactions, safety and effectiveness.

### 3.11 The bioavailability of nutmeg seed

Bioavailability highlights the efficiency of a constituent that enters the body and contribute in the biological activities. It refers to the level of a nutrient or drug, that reaches the bloodstream to initiate its proposed effects (Herkenne et al., 2008). The bioavailability of a substance, can be affected by route of administration, metabolism, its preparation and interactions with other materials (Olivares-Morales, et al., 2014). However, nutmeg bioavailability could be influenced by its metabolism pathway in the liver and the lipid constituents which may expand the lipophilic composites absorption (Zhao et al., 2020). Lipid has been found to accelerate the absorption of substances due to its impact on the P-glycoprotein (Markovic et al., 2020). Moreover, the nutmeg bioavailability can be affected by its interaction with food or medicines, which highlights the importance of nutrient-drug interactions in therapeutic applications (Stein et al., 2001). In a notable study, Sohn et al. found that the bioavailability of nutmeg lignan was 16.2 % after an intragastric administration of 60 mg/kg and an intravenous injection of 15 mg/kg. The concentration of nutmeg lignan remind high especially in the liver, and was quickly circulated to reach many body tissues. In addition, the bioavailability of nutmeg lignan has been found different for different administration routes. The bioavailability was 100 % of intravenous injection, while 25 % to 30 % oral route, with a ratio of 1 to 4 (Song et al., 2019). A full understanding of the bioavailability of nutmeg seed remains vague. Further studies are needed to provide valued perceptions about the bioavailability of nutmeg and its bioactive compounds. The use of in vivo models and other advanced analytical techniques could offer more path for future application. In summary, the insight of the bioavailability of nutmeg seed is essential to optimize its potential therapeutic use, and facilitate its development as a valuable nutraceutical or pharmaceutical agent.

### 3.12 Nutmeg seed in clinical trials

Clinical trials hold tremendous importance in drug development, as it helps to determine how effective is the drug use. Clinical trials of nutmeg are used to investigate the how the nutmeg utilized and functions in the body after being administrated via various routes. The investigation of nutmeg’s therapeutic potential in clinical trials is limited. Moreover, different methodologies have been applied to evaluate the nutmeg potency, efficacy, and safety on animals and cell lines. Recently, scientists showed their interest in using nutmeg in clinical trials. These clinical trials explained the multifaced pharmacological properties of nutmeg. In a recent clinical trial, Padol et al. (2022) demonstrated that nutmeg mouthwash effectively reduces dental biofilm build-up and halitosis. The attractive trait of this mouthwash is its origin source, since it is organic and relatively cost-effective compared with CHX, another normal mouthwash that was used in the trail. This organic alternative can replace 0.2 % CHX gluconate mouthwash, offering affordability and promoting oral hygiene, especially among those with limited financial means. A notable invention is on the way regarding the development of using nutmeg in dental care which is done by (Setty et al., 2022). They verified the application of nutmeg in primary teeth pulpotomy, revealed its efficacy as a pulpotomy agent, and demonstrating comparability to Mineral Trioxide Aggregate. Besides this, a recent clinical trials established that nutmeg in combination with garlic, onion, mango, black plum, and clove can lower lipid levels in hyperlipidemia patients (Alam et al., 2023). On the other hand, nonsignificant improvement in polyneuropathy disability (a hallmark of nerve damage) scores was recorded by Motilal and Maharaj (2013) when the topical nutmeg extract was used for 4 weeks. It is conceivable that clinical trials of nutmeg can provide significant perceptions about how the nutmeg interact within the body systems and its potential therapeutic implementations. Therefore, more investigations are needed to be done to evaluate nutmeg’s effect in preclinical and clinical trial studies in order to prove it efficacy.

## 4 Conclusion

The application of Nutmeg seed in the food and pharmaceutical industries arises as a promising natural substitute due to its ironic range of bioactive compounds with miscellaneous therapeutic properties. This review underlines the potential use of nutmeg as a complementary therapy with conventional treatments for countless illnesses, including microbial infections, inflammation, cancer, obesity, diabetes, and liver damage. Moreover, nutmeg's active compounds intensifying its therapeutic potential, and play a great role in promoting healthiness, beauty, and mental function. Therefore, future research focusing on human studies and clinical trials using nutmeg are more warranted in order to validate its therapeutic effects in treating various illnesses. On top of that, formulation and extraction methods optimization are essential in enhancing the bioavailability of nutmeg bioactive compounds and their effective dose. Investigating the targeted delivery and innovative applications of nutmeg-based medication beyond its traditional use is vital. Thus, toxicological studies are also needed to assess the nutmeg safety profile precisely, with a practical approach to clarify its potential therapeutic mechanisms. In addition, prolonged safety studies are also required to assess its compatibility for the management of chronic disease and to address its adverse effects. Tackling these studies approaches could deepen our perspectives and understanding of nutmeg's therapeutic potential and its assimilation into conventional healthcare applications.

**Table:** 

List of Abbreviations	
AG	Arachidonoylglycerol
AA	Arachidonic acid
ABTS	2,2'-Azino-bis (3-ethylbenzothiazoline-6-sulfonic acid)
BHT	Butylated hydroxytoluene
BSA	Bovine serum albumin
CDC	Centers for Disease Control and Prevention
CHX	Chlorhexidine
COVID-19	Coronavirus Disease 2019
COX-2	Cyclooxygenase-2
DPPH	2,2-diphenyl-1-picrylhydrazyl
dsRNA	Double-stranded RNA
E. coli	Escherichia coli
ECS	Endocannabinoid system
EFSA	European Food Safety Authority
FAAH	Fatty acid amide hydrolase
FAO	Food and Agriculture Organization
FRAP	Ferric-reducing antioxidant power
FTC	Ferrothiocyanate
GAE	Gallic acid equivalents
GC	Gas Chromatography
GCMS	Gas Chromatography-Mass Spectrometry
GCTOFMS	Gas Chromatography Time-of-Flight Mass Spectrometry
HPLC	High Performance Liquid Chromatography
IC50	Half maximal inhibitory concentration
IL-1β	Interleukin-1 Beta
IL-6	Interleukin-6
ISI	Integrated Taxonomic Information System
JAK-STAT	Janus Kinase-Signal Transducer and Activator of Transcription
LD50	Median lethal dose
LD50	Median lethal dose
MAGL	Monoacylglycerol lipase
MAPK	Mitogen-Activated Protein Kinase
MIC	Minimum Inhibitory Concentration
MRSA	Methicillin-Resistant Staphylococcus aureus
NF-κB	Nuclear Factor Kappa-Light-Chain-Enhancer of Activated B Cells
NIST	National Institute of Standards and Technology
NO	Nitric Oxide
NOS	Nitric Oxide Synthase
OSI	Oil stability index
PCR	Polymerase Chain Reaction
PADs	Microfluidic paper-based analytical devices
S. aureus	Staphylococcus aureus
SIRT1	Sirtuin 1
SK-N-SH	Human neuroblastoma cells
T. gondii	Toxoplasma gondii
TNF-α	Tumor Necrosis Factor Alpha
Unani	Traditional Greco-Arabic system of medicine
UTI	Urinary Tract Infection
WHO	World Health Organization

## CRediT authorship contribution statement

**Sawsan S. Al-Rawi:** Conceptualization, Data curation, Formal analysis, Funding acquisition, Investigation, Methodology, Software, Supervision, Resources, Validation, Visualization, Writing – original draft, Writing – review & editing. **Ahmad Hamdy Ibrahim:** Funding acquisition, Investigation, Methodology, Resources, Software, Validation, Visualization, Writing – original draft, Writing – review & editing. **Heshu Jalal Ahmed:** Validation, Writing – original draft, Writing – review & editing, Funding acquisition, Investigation, Resources. **Zhikal Omar Khudhur:** Funding acquisition, Resources, Validation, Writing – original draft, Writing – review & editing.

## Declaration of competing interest

The authors declare that they have no known competing financial interests or personal relationships that could have appeared to influence the work reported in this paper.

## References

[b0005] Ab Rahman N.N., Al-Rawi S.S., Ibrahim A.H., Nama M.M.B., Ab Kadir M.O. (2011). Supercritical carbon dioxide extraction of the residual oil from palm kernel cake. J. Food Eng..

[b0010] Abernethy M.K., Becker L.B. (1992). Acute nutmeg intoxication. Am. J. Emerg. Med..

[b0015] Adjene J.O., Nwose E.U. (2010). Histological effects of long-term consumption of nutmeg on the medial geniculate body of adult Wistar rats. N. Am. J. Med. Sci..

[b0020] Adwas A.A., Elsayed A., Azab A.E., Quwaydir F.A. (2019). Oxidative stress and antioxidant mechanisms in human body. J. Appl. Biotechnol. Bioeng.

[b0025] Ahmad S., Latif A., Qasmi I.A., Amin K.M.Y. (2005). An experimental study of sexual function improving effect of Myristica fragrans Houtt. (nutmeg). BMC Complement. Altern. Med..

[b0030] Ahmad H., Tijerina M.T., Tobola A.S. (1997). Preferential over expression of a class MU glutathione S-transferase subunit in mouse liver by myristicin. Biochem. Biophys. Res. Commun..

[b0035] Ahmed H.J., Ganjo A.R. (2019). Detection of carbapenemase-producing Klebsiella pneumoniae and Escherichia coli recovered from clinical specimens in Erbil City Kurdistan Region of Iraq. Al-Mustansiriyah Journal of Science.

[b0040] Aisyah Y., Yunita D., Amanda A. (2021). Paper Presented at the IOP Conference Series: Earth and Environmental Science.

[b0045] Akinwunmi K.F., Oyedapo O.O. (2014). In vitro anti-inflammatory evaluation of African nutmeg (Monodora myristica) seeds. Methodology.

[b0050] Al-Ajlouni A., Wesseling S., Soffers A.E., Al-Subeihi A., Kiwamoto R., Vervoort J., Rietjens I.M. (2017). Physiologically based kinetic modeling of the bioactivation of myristicin. Arch. Toxicol..

[b0055] Alam K., Sheikh H., Samad M.A. (2023). Clinical effect of poly herbal Unani formulation on dyslipidemia-a randomized trial. Turkish J. Agric.-Food Sci. Technol..

[b0060] Allegrante J.P., Wells M.T., Peterson J.C. (2019). Interventions to support behavioral self-management of chronic diseases. Annu. Rev. Public Health.

[b0065] Al-Qahtani W.H., Dinakarkumar Y., Arokiyaraj S., Saravanakumar V., Rajabathar J.R., Arjun K., Gayathri P.K., Appaturi J.N. (2022). Phyto-chemical and biological activity of Myristica fragrans, an ayurvedic medicinal plant in Southern India and its ingredient analysis. Saudi J. Biol. Sci..

[b0070] Al-Rawi S.S., Ibrahim A.H., Ab Rahman N.N.N., Nama M.M.B., Majid A.M.A., Ab Kadir M.O. (2011). The effect of supercritical fluid extraction parameters on the nutmeg oil extraction and its cytotoxic and antiangiogenic properties. Proc. Food Sci..

[b01182] Al-Rawi S.S., Ibrahim AH., Hamde MA., Babu D., Nazari M., Ab Kadir, Abdul Majid AS., Majid A.M.A. (2023). Antiangiogenic and Anticancer Potential of Supercritical Fluid Extracts from Nutmeg Seeds; In vitro, Ex vivo and in silico studies. Journal of Angiotherapy.

[b0075] Al-Rawi S.S., Ibrahim A.H., Majid A.S.A., Majid A.M.A., Ab Kadir M.O. (2013). Comparison of yields and quality of nutmeg butter obtained by extraction of nutmeg rind by soxhlet and supercritical carbon dioxide (SC-CO2). J. Food Eng..

[b0080] Al-Rawi S.S., Ibrahim A.H., Hamde M.A., Babu D., Nazari M., Ab Kadir M.O., Abdul Majid A.S., Majid A.M.A. (2023). Antiangiogenic and anticancer potential of supercritical fluid extracts from nutmeg seeds; In vitro, ex vivo and in silico studies. J. Angiotherapy.

[b0085] Al-Rawi S.S., Ibrahim A.H., Ahmed G.B. (2023). Unraveling the link between lymphopenia and systemic lupus erythematosus: implications for disease severity and potential treatment strategies. Eurasian J. Sci. Eng..

[b0090] Anand, P., 2008. Cancer is a Preventable Disease that Requires Major Lifestyle. https://www.ncbi.nlm.nih.gov › articles › PMC2515569. 10.1007/s11095-008-9661-9 PMC2515569 18626751

[b0095] Anwar H., Hussain G., Mustafa I. (2018). Antioxidants from natural sources. Antioxidants Foods Appl..

[b0100] Arshad H., Ali T.M., Abbas T., Hasnain A. (2018). Effect of microencapsulation on antimicrobial and antioxidant activity of nutmeg oleoresin using mixtures of gum arabic, osa, and native sorghum starch. Starch-Stärke.

[b0105] Arulmozhi D.K., Kurian R., Veeranjaneyulu A., Bodhankar S.L. (2007). Antidiabetic and antihyperlipidemic effects of myristica fragrans in animal models. Pharm. Biol..

[b0110] Asif M. (2015). Chemistry and antioxidant activity of plants containing some phenolic compounds. Chem. Int..

[b0115] Assa J.R., Widjanarko S.B., Kusnadi J., Berhimpon S. (2014). Antioxidant potential of flesh, seed and mace of nutmeg (Myristica fragrans Houtt). Int. J. Chem. Tech. Res..

[b0120] Azad N., Iyer A.K.V. (2014). System Biology of Free Radical and Antioxidants.

[b0125] Azis Ikhsanudin L.L., Rais D.D. (2021). Anti-inflammatory activity of Indonesian nutmeg seeds (Myristica fragrans Houtt): A topical gel formulation. Int. J. Public Health.

[b0130] Baker I., Chohan M., Opara E.I. (2013). Impact of cooking and digestion, in vitro, on the antioxidant capacity and anti-inflammatory activity of cinnamon, clove and nutmeg. Plant Foods Hum. Nutr..

[b0135] Balakrishnan S., Sivaji I., Kandasamy S., Duraisamy S., Kumar N.S., Gurusubramanian G. (2017). Biosynthesis of silver nanoparticles using Myristica fragrans seed (nutmeg) extract and its antibacterial activity against multidrug-resistant (MDR) Salmonella enterica serovar Typhi isolates. Environ. Sci. Pollut. Res..

[b0140] Barceloux D.G. (2009). Nutmeg (Myristica fragrans Houtt). Disease-a-Month. Foodborne and Microbial Toxins, Part II Staples and Spices.

[b0145] Beckerman B., Persaud H. (2019). Nutmeg overdose: spice not so nice. Complement. Ther. Med..

[b0150] Beyer J., Ehlers D., Maurer H.H. (2006). Abuse of nutmeg (Myristica fragrans Houtt.): studies on the metabolism and the toxicologic detection of its ingredients elemicin, myristicin, and safrole in rat and human urine using gas chromatography/mass spectrometry. Ther. Drug Monit..

[b0155] Bhutkar M.A., Somnath D.B., Dheeraj S.R., Ganesh H.W., Sachin S.T. (2018). In vitro studies on alpha amylase inhibitory activity of some indigenous plants. Modern Appl. Pharm. Pharmacol..

[b0160] Blokhina S.V., Ol'khovich M.V., Sharapova A.V., Levshin I.B., Perlovich G.L. (2021). Thermodynamic insights to solubility and lipophilicity of new bioactive hybrids triazole with thiazolopyrimidines. J. Mol. Liq..

[b0165] Brenner N., Frank O.S., Knight E. (1993). Chronic Nutmeg psychosis. J. R. Soc. Med..

[b0170] Broadhurst C.L., Polansky M.M., Anderson R.A. (2000). Insulin-like biological activity of culinary and medicinal plant aqueous extracts in vitro. J. Agric. Food Chem..

[b0175] Buckle J. (2014).

[b0180] Cao G.Y., Yang X.W., Xu W., Li F. (2013). New inhibitors of nitric oxide production from the seeds of Myristica fragrans. Food Chem. Toxicol..

[b0185] Cao G.Y., Xu W., Yang X.W., Gonzalez F.J., Li F. (2015). New neolignans from the seeds of Myristica fragrans that inhibit nitric oxide production. Food Chem..

[b0190] Casale M.J., MacDonald L.Q.L., Mitra A. (2023). Nutmeg Intoxication: A Case Report. Cureus.

[b0195] Centers for Disease Control and Prevention. July 7, (2022). What is Diabetes? Access from https://www.cdc.gov/diabetes/basics/diabetes.html.

[b0200] Chatterjee S., Niaz Z., Gautam S., Adhikari S., Variyar P.S., Sharma A. (2007). Antioxidant activity of some phenolic constituents from green pepper (Piper nigrum L.) and fresh nutmeg mace (Myristica fragrans). Food Chem..

[b0205] Chen L., Deng H., Cui H., Fang J., Zuo Z., Deng J. (2018). Inflammatory responses and inflammation-associated diseases in organs. Oncotarget.

[b0210] Chirathaworn C., Kongcharoensuntorn W., Dechdoungchan T., Lowanitchapat A., Sanguanmoo P., Poovorawan Y. (2007). Myristica fragrans Houtt. methanolic extract induces apoptosis in a human leukemia cell line through SIRT1 mRNA downregulation. J Med Assoc Thai..

[b0215] Choi E.J., Kang Y.G., Kim J., Hwang J.K. (2011). Macelignan inhibits melanosome transfer mediated by protease-activated receptor-2 in keratinocytes. Biol. Pharm. Bull..

[b0220] Chumkaew P., Srisawat T. (2019). New neolignans from the seeds of Myristica fragrans and their cytotoxic activities. J. Nat. Med..

[b0225] Cossetin L.F., Santi E.M., Garlet Q.I., Matos A.F., De Souza T.P., Loebens L. (2021). Comparing the efficacy of nutmeg essential oil and a chemical pesticide against Musca domestica and Chrysomya albiceps for selecting a new insecticide agent against synantropic vectors. Exp. Parasitol..

[b0230] Crozier A., Clifford M.N., Ashihara H. (2006).

[b0235] Dahham S.S., Al-Rawi S.S., Ibrahim A.H., Majid A.S.A., Majid A.M.S.A. (2018). Antioxidant, anticancer, apoptosis properties and chemical composition of black truffle Terfezia claveryi. Saudi J. Biol. Sci..

[b0240] de Souza B.B., Haute G.V., Ortega-Ribera M., Luft C., Antunes G.L., Bastos M.S. (2021). Methoxyeugenol deactivates hepatic stellate cells and attenuates liver fibrosis and inflammation through a PPAR-ɣ and NF-kB mechanism. J. Ethnopharmacol..

[b0245] Deng W., Du H., Liu D., Ma Z. (2022). Editorial: the role of natural products in chronic inflammation. Front. Pharmacol..

[b0250] Deng H., Li W. (2020). Monoacylglycerol lipase inhibitors: modulators for lipid metabolism in cancer malignancy, neurological and metabolic disorders. Acta Pharm. Sin. B.

[b0255] Desai C. (2016). Meyler's side effects of drugs: The international encyclopedia of adverse drug reactions and interactions. Indian J. Pharmacol..

[b0260] Devi M.T., Saha S., Tripathi A.M., Dhinsa K., Kalra S.K., Ghoshal U. (2019). Evaluation of the antimicrobial efficacy of herbal extracts added to root canal sealers of different bases: an in vitro study. Int. J. Clin. Pediatric Dentistry.

[b0265] Dewi K., Widyarto B., Erawijantari P.P., Widowati W. (2015). In vitro study of Myristica fragrans seed (Nutmeg) ethanolic extract and quercetin compound as anti-inflammatory agent. Int. J. Res. Med. Sci..

[b0270] Di X., Rouger C., Hardardottir I., Freysdottir J., Molinski T.F., Tasdemir D., Omarsdottir S. (2018). 6-Bromoindole derivatives from the Icelandic marine sponge Geodia barretti: Isolation and anti-inflammatory activity. Mar. Drugs.

[b0275] DiNatale B.C., Murray I.A., Schroeder J.C., Flaveny C.A., Lahoti T.S., Laurenzana E.M. (2010). Kynurenic acid is a potent endogenous aryl hydrocarbon receptor ligand that synergistically induces interleukin-6 in the presence of inflammatory signaling. Toxicol. Sci..

[b0280] Dkhil M.A., Abdel Moneim A.E., Hafez T.A., Mubaraki M.A., Mohamed W.F., Thagfan F.A., Al-Quraishy S. (2019). Myristica fragrans kernels prevent paracetamol-induced hepatotoxicity by inducing anti-apoptotic genes and Nrf2/HO-1 pathway. Int. J. Mol. Sci..

[b0285] Dogara A.M., Hamad S.W., Hama H.A., Bradosty S.W., Kayfi S., Al-Rawi S.S., Lema A.A. (2022). Biological evaluation of Garcinia kola Heckel. Adv. Pharm. Pharm. Sci..

[b0290] Dorman H.J., Deans S.G. (2000). Antimicrobial agents from plants: antibacterial activity of plant volatile oils. J. Appl. Microbiol..

[b0295] El-Alfy A.T., Joseph S., Brahmbhatt A., Akati S., Abourashed E.A. (2016). Indirect modulation of the endocannabinoid system by specific fractions of nutmeg total extract. Pharm. Biol..

[b0300] El-Alfy A.T., Abourashed E.A., Patel C., Mazhari N., An H., Jeon A. (2019). Phenolic compounds from nutmeg (Myristica fragrans Houtt.) inhibit the endocannabinoid-modulating enzyme fatty acid amide hydrolase. J. Pharm. Pharmacol..

[b0305] Eweka A.O., Eweka A. (2010). Histological effects of oral administration of nutmeg on the kidneys of adult Wister rats. N. Am. J. Med. Sci..

[b0310] Faisal S., Jan H., Shah S.A., Shah S., Khan A., Akbar M.T. (2021). Green synthesis of zinc oxide (ZnO) nanoparticles using aqueous fruit extracts of Myristica fragrans: their characterizations and biological and environmental applications. ACS Omega.

[b0315] Fernando, A., Senevirathne, W., 2021. Effect of essential oil of nutmeg (Myristica fragrans) leaves to treat human pathogenic bacteria and to manage plant pathogenic fungi. 10th Annual Science Research Session – FAS.

[b0320] Figueroa-Lopez K.J., Andrade-Mahecha M.M., Torres-Vargas O.L. (2018). Development of antimicrobial biocomposite films to preserve the quality of bread. Molecules.

[b0325] Food and Agriculture Organization of The United Nations, 1994. Nutmeg and derivatives. Rome, September. FO: MISC/94/7. Working Paper. Accessed on 10 May 2011, on line edition From the World Wide Web. http://www.fao.org/docrep/v4084e/v4084e00.htm#Contents.

[b0330] Francis T., Sankari Malaiappan D.S.R. (2022). Anti-inflammatory and cytotoxic effect of nutmeg based gel. J. Coastal Life Med..

[b0335] Freedman P. (2015). Health, wellness and the allure of spices in the Middle Ages. J. Ethnopharmacol..

[b0340] García-Díez J., Alheiro J., Falco V., Fraqueza M.J., Patarata L. (2017). Chemical characterization and antimicrobial properties of herbs and spices essential oils against pathogens and spoilage bacteria associated to dry-cured meat products. J. Essent. Oil Res..

[b0350] Gils C.V., Cox P.A. (1994). Ethnobotany of nutmeg in the Spice Islands. J. Ethnopharmacol..

[b0355] Ginting B., Mustanir M., Helwati H., Desiyana L.S., Eralisa E., Mujahid R. (2017). Antioxidant activity of n-hexane extract of nutmeg plants from South Aceh Province. J. Natural.

[b0360] Gordon A. (2020). Food Safety and Quality Systems in Developing Countries.

[b0365] Grover J.K., Khandkar S., Vats V., Dhunnoo Y., Das D. (2002). Pharmacological studies on Myristica fragrans–antidiarrheal, hypnotic, analgesic and hemodynamic (blood pressure) parameters. Methods Find. Exp. Clin. Pharmacol..

[b0370] Gupta A.D., Bansal V.K., Babu V., Maithil N. (2013). Chemistry, antioxidant and antimicrobial potential of nutmeg (Myristica fragrans Houtt). J. Genet. Eng. Biotechnol..

[b0375] Gupta S.D., Rao G.B., Bommaka M.K., Raghavendra N.M., Aleti S. (2016). Eco-sustainable synthesis and biological evaluation of 2-phenyl 1, 3-benzodioxole derivatives as anticancer, DNA binding and antibacterial agents. Arab. J. Chem..

[b0380] Hage C., Michaëlsson E., Linde C., Donal E., Daubert J.C., Gan L.M., Lund L.H. (2017). Inflammatory biomarkers predict heart failure severity and prognosis in patients with heart failure with preserved ejection fraction: a holistic proteomic approach. Circ. Cardiovasc. Genet..

[b0385] Hallström H., Thuvander A. (1997). Toxicological evaluation of myristicin. Nat Toxins.

[b0390] Han K.L., Choi J.S., Lee J.Y., Song J., Joe M.K., Jung M.H. (2008). Therapeutic potential of peroxisome proliferators–activated receptor-alpha/gamma dual agonist with alleviation of endoplasmic reticulum stress for the treatment of diabetes. Diabetes.

[b0395] Hanif M.A., Bhatti H.N., Jamil M.S., Anjum R.S., Jamil A., Khan M.M. (2010). Antibacterial and antifungal activities of essential oils extracted from medicinal plants using CO2 supercritical fluid extraction technology. Asian J. Chem..

[b0400] Hanif A., Ibrahim A.H., Ismail S., Al-Rawi S.S., Ahmad J.N., Hameed M. (2023). Cytotoxicity against A549 human lung cancer cell line via the mitochondrial membrane potential and nuclear condensation effects of Nepeta paulsenii Briq., a Perennial Herb. Molecules.

[b0405] Hartanto S., Ko H.S., Jee S.H., Kang J.U., Seo J.S., Kang Y.H. (2019). Effect of dietary nutmeg oil on heat-stress tolerance-related parameters in Korean native chicken reared under hot temperature. J. Anim. Physiol. Anim. Nutr..

[b0410] Hausner E.A., Poppenga R.H. (2012). Hazards associated with the use of herbal and other natural products. Small Animal Toxicology.

[b0415] Hemlata B., Pornima G., Tukaram K., Pankaj B. (2019). In vitro anti-amylase activity of some Indian dietary spices. J. Appl. Biol. Biotechnol..

[b0420] Herkenne C., Alberti I., Naik A., Kalia Y.N., Mathy F.X., Préat V., Guy R.H. (2008). In vivo methods for the assessment of topical drug bioavailability. Pharm. Res..

[b0425] Hoda S., Vermani M., Joshi R.K., Shankar J., Vijayaraghavan P. (2020). Anti-melanogenic activity of Myristica fragrans extract against Aspergillus fumigatus using phenotypic based screening. BMC Complementary Med. Therap..

[b0430] Holstege, C.P., 2005. Nutmeg. Encyclopedia of Toxicology (Second Edition): Blue Ridge Poison Center, Charlottesville, VA, USA, pp. 276-277.

[b0435] Horison R., Sulaiman F., Alfredo D., Wardana A. (2019). Physical characteristics of nanoemulsion from chitosan/nutmeg seed oil and evaluation of its coating against microbial growth on strawberry. Food Res..

[b0440] Hou J.-P., Wu H., Wang Y., Weng X.-C. (2012). Isolation of some compounds from nutmeg and their antioxidant activities. Czech J. Food Sci..

[b0445] Hwang J.K. (2010). Effects of macelignan isolated from Myristica fragrans Houtt. on UVB-induced matrix metalloproteinase-9 and cyclooxygenase-2 in HaCaT cells. J. Dermatol. Sci..

[b0450] Ibrahim A.H., Al-Rawi S.S. (2018).

[b0455] Ibrahim A.H., Al-Rawi S.S., Majid A.A., Rahman N.A., Abo-Salah K.M., Ab Kadir M.O. (2011). Separation and fractionation of Aquilaria malaccensis oil using supercritical fluid extraction and the cytotoxic properties of the extracted oil. Procedia Food Sci..

[b0460] Ibrahim M.A., Cantrell C.L., Jeliazkova E.A., Astatkie T., Zheljazkov V.D. (2020). Utilization of nutmeg (Myristica fragrans Houtt.) seed hydrodistillation time to produce essential oil fractions with varied compositions and pharmacological effects. Molecules.

[b0465] Ibrahim A.H., Li H., Al-Rawi S.S., Majid A.S.A., Al-Habib O.A., Xia X. (2017). Angiogenic and wound healing potency of fermented virgin coconut oil: in vitro and in vivo studies. Am. J. Transl. Res..

[b0470] Integrated Taxonomic Information System ITIS– Myristica fragrans Houtt Report. (2023). https://www.itis.gov/servlet/SingleRpt/SingleRpt?search_topic=TSN&search_value=18125#null.

[b0475] International Trade Centre (ITC) (2003).

[b0480] Islam M.S. (2022). Natural products and disease prevention, relief and treatment. Nutrients.

[b0485] Iwata N., Kobayashi D., Kawashiri T., Kubota T., Kawano K., Yamamuro Y. (2022). Mechanisms and safety of antidepressant-like effect of nutmeg in mice. Biol. Pharm. Bull..

[b0490] Jaiswal Y.S., Williams L.L. (2017). A glimpse of Ayurveda-The forgotten history and principles of Indian traditional medicine. J. Tradit. Complement. Med..

[b0495] Jalal Ahmed H., Ibrahim H.A., Al-Rawi S., Ganjo R.A., Fryad Saber H. (2023). Molecular Characterization of Carbapenem resistant Escherichia coli and Klebsiella pneumoniae in Erbil, Iraq. J. Popul. Ther. Clin. Pharmacol..

[b0500] Jan M., Faqir F., Hamida M.MA. (2005). Comparison of effects of extract of Myristica fragrans and verapamil on the volume and acidity of carbachol induced gastric secretion in fasting rabbits. J. Ayub Med. College, Abbottabad: JAMC..

[b0505] Jang D.I., Lee A.H., Shin H.Y., Song H.R., Park J.H., Kang T.B. (2021). The role of tumor necrosis factor alpha (TNF-α) in autoimmune disease and current TNF-α inhibitors in therapeutics. Int. J. Mol. Sci..

[b0510] Kapoor I., Singh B., Singh G., De Heluani C.S., De Lampasona M., Catalan C.A. (2013). Chemical composition and antioxidant activity of essential oil and oleoresins of nutmeg (Myristica fragrans Houtt.) fruits. Int. J. Food Prop..

[b0515] Kareem M.A., Gadhamsetty S.K., Shaik A.H., Prasad E.M., Kodidhela L.D. (2013). Protective effect of nutmeg aqueous extract against experimentally induced hepatotoxicity and oxidative stress in rats. J. Ayurveda Integrative Med..

[b0520] Kasatkina L.A., Rittchen S., Sturm E.M. (2021). Neuroprotective and immunomodulatory action of the endocannabinoid system under neuroinflammation. Int. J. Mol. Sci..

[b0525] Kazeem M., Akanji M., Hafizur R.M., Choudhary M. (2012). Antiglycation, antioxidant and toxicological potential of polyphenol extracts of alligator pepper, ginger and nutmeg from Nigeria. Asian Pac. J. Trop. Biomed..

[b0530] Kazlauskaite J.A., Matulyte I., Marksa M., Bernatoniene J. (2023). Nutmeg Essential Oil, Red Clover, and Liquorice Extracts Microencapsulation Method Selection for the Release of Active Compounds from Gel Tablets of Different Bases. Pharmaceutics.

[b0535] Kiarsi Z., Hojjati M., Behbahani B.A., Noshad M. (2020). In vitro antimicrobial effects of Myristica fragrans essential oil on foodborne pathogens and its influence on beef quality during refrigerated storage. J. Food Saf..

[b0540] Kim, H. J., Chen, F., Wang, X., Wang, Y., McGregor, J., Jiang, Y. M., 2010. Characterization of antioxidants in nutmeg (Myristica fragrans Houttuyn) oil. In: *Flavor and Health Benefits of Small Fruits.* American Chemical Society, pp. 239-252.

[b0545] Kim J.H., Kismali G., Gupta S.C. (2018). Natural Products for the Prevention and Treatment of Chronic Inflammatory Diseases: Integrating Traditional Medicine into Modern Chronic Diseases Care. Evidence-Based Complementary Alternative Med..

[b0550] Kimura Y., Ito H., Hatano T. (2010). Effects of mace and nutmeg on human cytochrome P450 3A4 and 2C9 activity. Biol. Pharm. Bull..

[b0555] Laird B.J., Kaasa S., McMillan D.C., Fallon M.T., Hjermstad M.J., Fayers P., Klepstad P. (2013). Prognostic Factors in Patients with Advanced Cancer: A Comparison of Clinicopathological Factors and the Development of an Inflammation-Based Prognostic SystemPrognostic Factors in Advanced Cancer. Clin. Cancer Res..

[b0560] Lee H.H., Jang E., Kang S.Y., Shin J.S., Han H.S., Kim T.W. (2020). Anti-inflammatory potential of Patrineolignan B isolated from Patrinia scabra in LPS-stimulated macrophages via inhibition of NF-κB, AP-1, and JAK/STAT pathways. Int. Immunopharmacol..

[b0565] Lee H.S., Jeong T.C., Kim J.H. (1998). In vitro and in vivo metabolism of myristicin in the rat. J. Chromatogr. B Biomed. Sci. Appl..

[b0570] Lee B.K., Kim J.H., Jung J.W., Choi J.W., Han E.S., Lee S.H., Koc K.H., Ryu J.H. (2005). Myristicin-induced neurotoxicity in human neuroblastoma SK-N-SH cells. Toxicol. Lett..

[b0575] Lee K.E., Mun S., Pyun H.B., Kim M.S., Hwang J.K. (2012). Effects of macelignan isolated from Myristica fragrans (Nutmeg) on expression of matrix metalloproteinase-1 and type I procollagen in UVB-irradiated human skin fibroblasts. Biol. Pharm. Bull..

[b0580] Lee J.Y., Park W. (2011). Anti-inflammatory effect of myristicin on RAW 264.7 macrophages stimulated with polyinosinic-polycytidylic acid. Molecules.

[b0585] Lei S., Zheng R., Zhang S., Wang S., Chen R., Sun K. (2021). Global patterns of breast cancer incidence and mortality: A population-based cancer registry data analysis from 2000 to 2020. Cancer Commun..

[b0590] Li X., Chen S., Zhang L., Niu G., Zhang X., Yang L. (2022). Coinfection of Porcine Circovirus 2 and Pseudorabies Virus Enhances Immunosuppression and Inflammation through NF-κB, JAK/STAT, MAPK, and NLRP3 Pathways. Int. J. Mol. Sci..

[b0595] Lim G.C.C. (2002). Overview of cancer in Malaysia. Jpn. J. Clin. Oncol..

[b0600] Liu T., Yan T., Jia X., Liu J., Ma R., Wang Y. (2022). Systematic exploration of the potential material basis and molecular mechanism of the Mongolian medicine Nutmeg-5 in improving cardiac remodeling after myocardial infarction. J. Ethnopharmacol..

[b0610] Loizzo M.R., Sicari V., Tenuta M.C., Leporini M.R., Falco T., Pellicanò T.M. (2016). Phytochemicals content, antioxidant and hypoglycaemic activities of commercial nutmeg mace (Myristica fragrans L.) and pimento (Pimenta dioica (L.) Merr.). Int. J. Food Sci. Technol..

[b0615] Long J., Qian K., Tan S., Liu J., Li J. (2020). Macelignan protects against renal ischemia-reperfusion injury via inhibition of inflammation and apoptosis of renal epithelial cells. Cellular and molecular biology (Noisy-le-Grand. France).

[b0620] López-Pedrouso M., Lorenzo J.M., Franco D. (2022). Advances in natural antioxidants for food improvement. Antioxidants.

[b0625] Lu J., Hu Y., Wang L., Wang Y., Na S., Wang J. (2018). Understanding the Multitarget Pharmacological Mechanism of the Traditional Mongolian Common Herb Pair GuangZao-RouDouKou Acting on Coronary Heart Disease Based on a Bioinformatics Approach. Evidence-Based Complementary Alternative Med.: eCAM..

[b0630] Machmudaha S., Sulaswatty A., Sasaki M., Goto M., Hirose T. (2006). Supercritical CO2 extraction of nutmeg oil: Experiments and modeling. J. of Supercritical Fluids.

[b0635] Mahady G.B., Pendland S.L., Stoia A., Hamill F.A., Fabricant D., Dietz B.M., Chadwick L.R. (2005). In Vitro susceptibility of Helicobacter pylori to botanical extracts used traditionally for the treatment of gastrointestinal disorders. Phytother. Res..

[b0640] Manier S.K., Wagmann L., Weber A.A., Meyer M.R. (2021). Abuse of nutmeg seeds: Detectable by means of liquid chromatography-mass spectrometry techniques?. Drug Test. Anal..

[b0645] Manohar M., Kandikattu H.K., Verma A.K., Mishra A. (2018). IL-15 regulates fibrosis and inflammation in a mouse model of chronic pancreatitis. American Journal of Physiology-Gastrointestinal and Liver. Physiology.

[b0650] Markovic M., Ben-Shabat S., Aponick A., Zimmermann E.M., Dahan A. (2020). Lipids and lipid-processing pathways in drug delivery and therapeutics. Int. J. Mol. Sci..

[b0655] Martins C., Doran C., Silva I.C., Miranda C., Rueff J., Rodrigues A.S. (2014). Myristicin from nutmeg induces apoptosis via the mitochondrial pathway and down regulates genes of the DNA damage response pathways in human leukaemia K562 cells. Chem. Biol. Interact..

[b0660] Matulyte I., Marksa M., Ivanauskas L., Kalvėnienė Z., Lazauskas R., Bernatoniene J. (2019). GC-MS analysis of the composition of the extracts and essential oil from Myristica fragrans seeds using magnesium aluminometasilicate as excipient. Molecules.

[b0665] Matulyte I., Jekabsone A., Jankauskaite L., Zavistanaviciute P., Sakiene V., Bartkiene E. (2020). The essential oil and hydrolats from Myristica fragrans seeds with magnesium aluminometasilicate as excipient: antioxidant, antibacterial, and anti-inflammatory activity. Foods.

[b0670] Maya K.M., Zachariah T.J., Krishnamoorthy B. (2004). Chemical composition of essential oil of nutmeg (Myristica fragrans Houtt.) accessions. Journal of Spices and Aromatic. Crops.

[b0675] McKenna A., Nordt S.P., Ryan J. (2004). Acute nutmeg poisoning. European J. Emergency Med..

[b0680] Mickus R., Jančiukė G., Raškevičius V., Mikalayeva V., Matulytė I., Marksa M. (2021). The effect of nutmeg essential oil constituents on Novikoff hepatoma cell viability and communication through Cx43 gap junctions. Biomed. Pharmacother..

[b0685] Modzelewska A., Sur S., Kumar S.K., Khan S.R. (2005). Sesquiterpenes: natural products that decrease cancer growth. Current Med. Chem. - Anti-Cancer Agents.

[b0690] Monahan K.J., Bradshaw N., Dolwani S., Desouza B., Dunlop M.G., East J.E. (2020). Guidelines for the management of hereditary colorectal cancer from the British Society of Gastroenterology (BSG)/Association of Coloproctology of Great Britain and Ireland (ACPGBI)/United Kingdom Cancer genetics group (UKCGG). Gut.

[b0695] Morita T., Jinno K., Kawagishi H., Arimoto Y., Suganuma H., Inakuma T., Sugiyama K. (2003). Hepatoprotective effect of myristicin from nutmeg (Myristica fragrans) on lipopolysaccharide/ D-galactosamine-induced liver injury. J. Agric. Food Chem..

[b0700] Morsy N.F. (2016). A comparative study of nutmeg (Myristica fragrans Houtt.) oleoresins obtained by conventional and green extraction techniques. J. Food Sci. Technol..

[b0705] Motilal S., Maharaj R.G. (2013). Nutmeg extracts for painful diabetic neuropathy: a randomized, double-blind, controlled study. J. Altern. Complement. Med..

[b0710] Moussa Z., Judeh Z.M., Ahmed S.A. (2019). Nonenzymatic exogenous and endogenous antioxidants. Free Rad. Med. Biol..

[b0715] Muchtaridi A.S., Apriyantono A., Mustarichie R. (2010). Identification of Compounds in the Essential Oil of Nutmeg Seeds (Myristica fragrans Houtt. That Inhibit Locomotor Activity in Mice. Int. J. Mol. Sci..

[b0720] Mulvihill M.M., Nomura D.K. (2013). Therapeutic potential of monoacylglycerol lipase inhibitors. Life Sci..

[b0725] Murcia M.A., Egea I., Romojaro F., Parras P., Jimenez A.M., Martinez-Tome M. (2004). Antioxidant evaluation in dessert spices compared with common food additives. Influence of irradiation procedure. J. Agric. Food Chem..

[b0730] Nagano I. (2009). Myristica fragrans: An Exploration of the Narcotic Spice. The Entheogen Review. Vernal Equinox.

[b0735] Nasreen W., Sarker S., Sufian M.A., Md Opo F.A.D., Shahriar M., Akhter R. (2020). A possible alternative therapy for type 2 diabetes using Myristica fragrans Houtt in combination with glimepiride: in vivo evaluation and in silico support. Z. Naturforsch. [C].

[b0740] National Institute of Standards and Technology, 2023. NIST Chemistry WebBook. Retrieved August 3, 2023 from https://webbook.nist.gov/chemistry/.

[b0745] Neukamm M.A., Schwelm H.M., Vieser S., Schiesel N., Auwärter V. (2020). Detection of nutmeg abuse by gas chromatography—Mass spectrometric screening of urine. J. Anal. Toxicol..

[b0750] Nguyen P.H., Le T.V.T., Kang H.W., Chae J., Kim S.K., Kwon K.I. (2010). AMP-activated protein kinase (AMPK) activators from Myristica fragrans (nutmeg) and their anti-obesity effect. Bioorg. Med. Chem. Lett..

[b0755] Nikolic V., Nikolic L., Dinic A., Gajic I., Urosevic M., Stanojevic L. (2021). Chemical composition, antioxidant, and antimicrobial activity of nutmeg (Myristica fragrans Houtt.) seed essential oil. J. Essential Oil Bearing Plants.

[b0760] Noguchi N., Niki E. (2019). Antioxidant Status, Diet, Nutrition, and Health.

[b0765] Obranović M., Bryś J., Repajić M., Balbino S., Škevin D., Bryś A. (2020). Fatty acid and sterol profile of nutmeg (Myristica fragrans) and star anise (Illicium verum) extracted using three different methods. Multidisciplinary Digital Publishing Institute Proceedings.

[b0770] Ogawa K., Ito M. (2019). Appetite-enhancing effects of nutmeg oil and structure–activity relationship of habituation to phenylpropanoids. J. Nat. Med..

[b0775] Okiki P.A., Nwobi C.P., Akpor O.B., Adewole E., Agbana R.D. (2023). Assessment of nutritional and medicinal properties of nutmeg. Scientific African.

[b0780] Olaleye M.T., Akinmoladun A.C., Akindahunsi A.A. (2006). Antioxidant properties of Myristica fragrans (Houtt) and its effect on selected organs of albino rats. Afr. J. Biotechnol..

[b0785] Olivares-Morales A., Hatley O.J., Turner D., Galetin A., Aarons L., Rostami-Hodjegan A. (2014). The use of ROC analysis for the qualitative prediction of human oral bioavailability from animal data. Pharm. Res..

[b0790] Onetto, A.J., Sharif, S., 2023. Drug Distribution. In: StatPearls [Internet]. Treasure Island (FL): StatPearls Publishing; 2024 Jan–. PMID: 33620813. 33620813

[b0795] Ongtanasup T., Wanmasae S., Srisang S., Manaspon C., Net-Anong S., Eawsakul K. (2022). In silico investigation of ACE2 and the main protease of SARS-CoV-2 with phytochemicals from Myristica fragrans (Houtt.) for the discovery of a novel COVID-19 drug. Saudi J. Biol. Sci..

[b0800] Onyenibe N.S., Fowokemi K.T., Emmanuel O.B. (2015). African Nutmeg (Monodora Myristica) Lowers Cholesterol and Modulates Lipid Peroxidation in Experimentally Induced Hypercholesterolemic Male Wistar Rats. Int. J. Biomed. Sci..

[b0805] Oo T., Saiboonjan B., Srijampa S., Srisrattakarn A., Sutthanut K., Tavichakorntrakool R., Tippayawat P. (2021). Inhibition of bacterial efflux pumps by crude extracts and essential oil from Myristica fragrans Houtt. (Nutmeg) seeds against methicillin-resistant Staphylococcus aureus. Molecules.

[b0810] Orabi M., Abdulsattar J.O., Nasi Z.O. (2022). Phytochemical Profile, Antimicrobial, Antioxidant Activity and Cyclooxygenase 2 Inhibitory Properties of Nutmeg (Myristica Fragrans) Seeds Extract. Egypt. J. Chem..

[b0815] Ostro B., Malig B., Broadwin R., Basu R., Gold E.B., Bromberger J.T. (2014). Chronic PM2. 5 exposure and inflammation: determining sensitive subgroups in mid-life women. Environ. Res..

[b0820] Oswald E.S., Fishbein L., Corbett B.J., Walker M.P. (1971). Urinary excretion of tertiary amino methoxy methylenedioxy propiophenones as metabolites of myristicin in the rat and guinea pig. Biochim. Biophys. Acta (BBA)-General Subjects.

[b0825] Özkan O.E., Olgun Ç., Güney B., Mahmut G.Ü.R., Güney K., Saim A.T.E.Ş. (2018). Chemical composition and antimicrobial activity of Myristica fragrans & Elettaria cardamomum essential oil. Kastamonu University J. Forestry Faculty.

[b0830] Padol M.V., Vishwakarma P., Dodamani A.S., Gore A.W., Chachlani K.S., Kharkar S.P. (2022). Comparative evaluation of nutmeg mouthwash and 0.2% chlorhexidine gluconate mouthwash on halitosis and plaque control: A randomized clinical trial. J. Indian Soc. Periodontol..

[b0835] Parle M., Dhingra D., Kulkarni S.K. (2004). Improvement of mouse memory by Myristica fragrans seeds. J. Med. Food.

[b0840] Parthasarathy V.A., Chempakam B., Zachariah T.J. (2008).

[b0845] Parvin R., Seo J.K., Eom J.U., Ahamed Z., Yang H.S. (2023). Inhibitory and antioxidative capacity of nutmeg extracts on reduction of lipid oxidation and heterocyclic amines in pan-roasted beef patties. Meat Sci..

[b0850] Pashapoor A., Mashhadyrafie S., Mortazavi P. (2020). Ameliorative effect of Myristica fragrans (nutmeg) extract on oxidative status and histology of pancreas in alloxan induced diabetic rats. Folia Morphol..

[b0855] Pauline M.C., Sangeetha R., Manikandan M., Loganathan P., Kalaiarasi J. (2019). Myristica Fragrans (Nutmeg) Oil Mediated Silver Nanoparticle Synthesis, Characterisation And Its Antimicrobial Assessment. Uttar Pradesh J. Zool..

[b0860] Pawar, N., 2023. Nutmeg. Encyclopedia of Toxicology (Fourth Edition), 7, 5-8. Academic Press, Fd.

[b0865] Payne R.B. (1963). Nutmeg intoxication. N. Engl. J. Med..

[b0870] Pereira A.S., Banegas-Luna A.J., Peña-García J., Pérez-Sánchez H., Apostolides Z. (2019). Evaluation of the anti-diabetic activity of some common herbs and spices: Providing new insights with inverse virtual screening. Molecules.

[b0875] Perumalsamy R., Krishnadhas L. (2022). Anti-Diabetic Activity of Silver Nanoparticles Synthesized from the Hydroethanolic Extract of Myristica fragrans Seeds. Appl. Biochem. Biotechnol..

[b0880] Pham V.C., Jossang A., Sévenet T., Bodo B. (2000). Cytotoxic acylphenols from Myristica maingayi. Tetrahedron.

[b0885] Piras A., Rosa A., Marongiu B., Atzeri A., Dessì M.A., Falconieri D., Porcedda S. (2012). Extraction and separation of volatile and fixed oils from seeds of Myristica fragrans by supercritical CO2: Chemical composition and cytotoxic activity on Caco-2 cancer cells. J. Food Sci..

[b0890] Prabha B., Sini S., Sherin D.R., Neethu S., Rameshkumar K.B., Manojkumar T.K. (2021). Promalabaricone B from Myristica fatua Houtt. seeds demonstrate antidiabetic potential by modulating glucose uptake via the upregulation of AMPK in L6 myotubes. Nat. Prod. Res..

[b0895] Pranati T., Anitha R., Rajeshkumar S., Lakshmi T. (2019). Preparation of silver nanoparticles using nutmeg oleoresin and its antimicrobial activity against oral pathogens. Res. J. Pharm. Technol..

[b0900] Pratiwi Y.S., Lesmana R., Goenawan H., Sylviana N., Setiawan I., Tarawan V.M. (2018). Nutmeg Extract Increases Skeletal Muscle Mass in Aging Rats Partly via IGF1-AKT-mTOR Pathway and Inhibition of Autophagy. Evid. Based Complement. Alternat. Med..

[b0905] Private Sector CARICOM’s nutmeg trade, 2009. Private Sector trade note of the Office of Trade Negotiations (OTN). Retrieved on 16 May 2011. On line edition From the World Wide Web: http://www.crnm.org/index.php?option=com_docman&task=cat_view&gid=98&Itemid=109.

[b0910] Purba, H. J., Yusufi, E. S., Hestina, J., 2021. Performane and competitiveness of indonesian nutmeg in export market. In *E3S Web of Conferences*, EDP Sciences, Vol. 232, p. 02018.

[b0915] Radzali S.A., Markom M., Md Saleh N. (2022). Parameter Effects and Optimisation in Supercritical Fluid Extraction of Phenolic Compounds from Labisia pumila. Separations.

[b0920] Raghupathi, W., Raghupathi, V., 2018. An Empirical Study of Chronic Diseases in the United States: (2018). A Visual Analytics Approach. Int. J. Environ. Res. Public Health. 15(3). 10.3390/ijerph15030431 PMC5876976 29494555

[b0925] Ram A., Lauria P., Gupta R., Sharma V.N. (1996). Hypolipidaemic effect of Myristica fragrans fruit extract in rabbits. J. Ethnopharmacol..

[b0930] Rancy A.T., Krishnakumari S. (2015). Phytochemical profiling of Myristica fragrans seed extract with different organic solvents. Asian J Pharma Clin Res.

[b0935] Rosmalia D., Marjoni M.R. (2022). Effect of Nutmeg (Myristica Fragrans) Methanolic Extract to the Growth of Dental Plaque Bacteria. DENTA.

[b0940] Roy A., Khan A., Ahmad I., Alghamdi S., Rajab B.S., Babalghith A.O. (2022). Flavonoids a Bioactive Compound from Medicinal Plants and Its Therapeutic Applications. Biomed Res. Int..

[b0945] Saleh M., Nabil Z., Mekkawy H., Abd A.G. (1989). Acute and chronic effects of a nutmeg extract on the toad heart. Pharmacol. Biochem. Behav.

[b0950] Salehi B., Machin L., Monzote L., Sharifi-Rad J., Ezzat S.M., Salem M.A. (2020). Therapeutic Potential of Quercetin: New Insights and Perspectives for Human Health. ACS Omega.

[b0955] Sarifah N., Indira Lanti P., Dwi Pretti S. (2017). Antibacterial Activity of Nutmeg Oil. KnE. Life Sci..

[b0960] Sattar A., Abdo A., Mushtaq M.N., Anjum I., Anjum A. (2019). Evaluation of Gastro-protective Activity of Myristica fragrans on Ethanol-induced Ulcer in Albino Rats. Anais Da Academia Brasileira De Ciencias..

[b0965] Setty J.V., Srinivasan I., Sathiesh R.T., Milit Y. (2022). Evaluation of the efficacy of Myristica Fragrans as a pulpotomy medicament in primary molars: A Clinical Trial. RGUHS. J. Dental Sci..

[b0970] Sharma A., Mathur R., Dixit V.P. (1995). Prevention of hypercholesterolemia and atherosclerosis in rabbits after supplementation of Myristica fragrans seed extract. Indian J. Physiol. Pharmacol..

[b0975] Smith M. (2014).

[b0980] Sohn J.H., Han K.L., Choo J.H., Hwang J.K. (2007). Macelignan protects HepG2 cells against tert-butylhydroperoxide-induced oxidative damage. BioFactors (oxford, England)..

[b0985] Song Y., Zhang Y., Duan X.Y., Cui D.W., Qiu X., Bian Y. (2019). Pharmacokinetics and tissue distribution of anwuligan in rats after intravenous and intragastric administration by liquid chromatography-mass spectrometry. Molecules.

[b0990] Spricigo C.B., Pinto L.T., Bolzan A., Novais A.F. (1999). Extraction of essential oil and lipids from nutmeg by liquid carbon dioxide. J. Supercrit. Fluids.

[b0995] Stein U., Greyer H., Hentschel H. (2001). Nutmeg (myristicin) poisoning—report on a fatal case and a series of cases recorded by a poison information centre. Forensic Sci. Int..

[b1000] Subaddarage J.S., Jansz E.R., Dharmadasa H.M. (1985). Some Physical and Chemical Characteristics of Sri Lankan Nutmeg Oil. J. Sci. Food Agric..

[b1005] Sumarni W., Sudarmin S., Sumarti S.S. (2019).

[b1010] Susianti S., Lesmana R., Salam S., Julaeha E., Pratiwi Y.S., Sylviana N. (2021). the effect of nutmeg seed (M. Fragrans) extracts induces apoptosis in melanoma maligna cell’s (B16–F10). Indonesian Biomed. J..

[b1015] Sylvester C. (2018). Izah., et al. Antibacterial Efficacy of Aqueous Extract of Myristica fragrans (Common Nutmeg). EC Pharmacol. Toxicol..

[b1020] Tajuddin A.S., Latif A., Qasmi I.A., Amin K.M. (2005). An experimental study of sexual function improving effect of Myristica fragrans Houtt (nutmeg). BMC Complementary Alternative Med..

[b1025] Takikawa A., Abe K., Yamamoto M., Ishimaru S., Yasui M., Okubo Y., Yokoigawa K. (2002). Antimicrobial activity of nutmeg against Escherichia coli O157. J. Biosci. Bioeng..

[b1030] Thileepan T., Thevanesam V., Kathirgamanathar S. (2017). Antimicrobial Activity of Seeds and Leaves of Myristica fragrans against Multi-resistant Microorganisms. J. Agric. Sci. Technol. A.

[b1035] Tomaino A., Cimino F., Zimbalatti V., Venuti V., Sulfaro V., De Pasquale A., Saija A. (2005). Influence of heating on antioxidant activity and the chemical composition of some spice essential oils. J Food Chemistry.

[b1040] Torres J., Enríquez-de-Salamanca A., Fernández I., Rodríguez-Ares M.T., Quadrado M.J., Murta J. (2011). Activation of MAPK signaling pathway and NF-κB activation in pterygium and ipsilateral pterygium-free conjunctival specimens. Invest. Ophthalmol. Vis. Sci..

[b1045] Tripathi I.P., Dwivedi N. (2015). Pharmacognostical standardization of nutmeg seeds (Myristica fragrans Houtt.)–a traditional medicine. Int J Pharm Sci Res.

[b1050] Tsai D.H., Riediker M., Berchet A., Paccaud F., Waeber G., Vollenweider P., Bochud M. (2019). Effects of short-and long-term exposures to particulate matter on inflammatory marker levels in the general population. Environ. Sci. Pollut. Res..

[b1055] Umayah S., Marhaendro P. (2021). The uniqueness of isolation of nutmeg essential oil from nutmeg seeds (Myristica fragrans Houtt.) and its effects on physical and chemical properties. Indian J. Nat. Prod. Resour..

[b1065] Usui K., Kubota E., Kobayashi H., Fujita Y., Hatanaka K., Kamijo Y. (2023). Detection of major psychoactive compounds (safrole, myristicin, and elemicin) of nutmeg in human serum via GC–MS/MS using MonoSpin® extraction: Application in a nutmeg poisoning case. J. Pharm. Biomed. Anal..

[b1070] Wagan T.A., Wang W., Hua H., Cai W. (2017). Chemical constituents and toxic, repellent, and oviposition-deterrent effects of ethanol-extracted Myristica fragrans (Myristicaceae) oil on Bemisia tabaci (Hemiptera: Aleyrodidae). Fla. Entomol..

[b1075] Weil A.T. (1971). Nutmeg as a psychoactive drug. J. Psychedelic Drugs.

[b1080] Wibowo D.P., Febriana Y., Riasari H., Auilifa D.L. (2018). Essential oil composition, antioxidant and antibacterial activities of nutmeg (Myristica fragrans Houtt) from Garut West Java. Indonesian J. Pharm. Sci. Technol..

[b1085] Wu Y., Hao C., Han G., Liu X., Xu C., Zou Z. (2021). SS-31 ameliorates hepatic injury in rats subjected to severe burns plus delayed resuscitation via inhibiting the mtDNA/STING pathway in Kupffer cells. Biochem. Biophys. Res. Commun..

[b1090] Wu N., Xu W., Cao G.Y., Yang Y.F., Yang X.B., Yang X.W. (2016). The blood-brain barrier permeability of lignans and malabaricones from the seeds of Myristica fragrans in the MDCK-pHaMDR cell monolayer model. Molecules.

[b1095] Xia W., Cao Z., Zhang X., Gao L. (2021). A proteomics study on the mechanism of nutmeg-induced hepatotoxicity. Molecules.

[b1100] Yakaiah V., Dakshinamoorthi A., Ty S.S. (2021). Novel Aspects in Inhibiting Pancreatic Lipase with Potential New Compound from Nutmeg in Connection with Obesity–In Vitro, In Silico, In Vivo and Ex Vivo Studies. Maedica.

[b1105] Yan T., Zhu X., Zhang X., Jia X., Liu J., Wang X. (2022). The application of proteomics and metabolomics to reveal the molecular mechanism of Nutmeg-5 in ameliorating cardiac fibrosis following myocardial infarction. Phytomed.: Int. J. Phytotherapy Phytopharmacol..

[b1110] Yang A.H., He X., Chen J.X., He L.N., Jin C.H., Wang L.L. (2015). Identification and characterization of reactive metabolites in myristicin-mediated mechanism-based inhibition of CYP1A2. Chem. Biol. Interact..

[b1115] Yang X.N., Liu X.M., Fang J.H., Zhu X., Yang X.W., Xiao X.R. (2018). PPARα mediates the hepatoprotective effects of nutmeg. J. Proteome Res..

[b1120] Yang S., Na M.K., Jang J.P., Kim K.A., Kim B.Y., Sung N.J. (2006). Inhibition of protein tyrosine phosphatase 1B by lignans from Myristica fragrans. Phytotherapy Research: PTR..

[b1125] Yang P.W., Xu P.L., Cheng C.S., Jiao J.Y., Wu Y., Dong S. (2022). Integrating network pharmacology and experimental models to investigate the efficacy of QYHJ on pancreatic cancer. J. Ethnopharmacol..

[b1130] Yoshioka Y., Kono R., Kuse M., Yamashita Y., Ashida H. (2022). Phenylpropanoids and neolignans isolated from Myristica fragrans enhance glucose uptake in myotubes. Food Funct..

[b1135] Yuan Z.M., Wang J., Lv J., Jia T.Z. (2006). Comparing analysis of components in volatile oils of nutmeg and prepared nutmeg by GC-MS. China J. Chinese Materia Medica.

[b1140] Yun C.H., Lee H.S., Lee H.Y., Yim S.K., Kim K.H., Kim E. (2003). Roles of human liver cytochrome P450 3A4 and 1A2 enzymes in the oxidation of myristicin. Toxicol. Lett..

[b1145] Zhang, Y.J., Gan, R.Y., Li, S., Zhou, Y., Li, A.N., Xu, D.P., et al., 2015. Antioxidant Phytochemicals for the Prevention and Treatment of Chronic Diseases. Molecules (Basel, Switzerland) 20(12), 21138. 10.3390/molecules201219753 PMC6331972 26633317

[b1150] Zhang J., Si H., Li B., Zhou X., Zhang J. (2019). Myrislignan exhibits activities against Toxoplasma gondii RH strain by triggering mitochondrial dysfunction. Front. Microbiol..

[b1155] Zhang W.K., Tao S.S., Li T.T., Li Y.S., Li X.J., Tang H.B. (2016). Nutmeg oil alleviates chronic inflammatory pain through inhibition of COX-2 expression and substance P release in vivo. Food Nutr. Res..

[b1160] Zhao M., Ma J., Li M., Zhang Y., Jiang B., Zhao X. (2021). Cytochrome P450 enzymes and drug metabolism in humans. Int. J. Mol. Sci..

[b1165] Zhao W., Song F., Hu D., Chen H., Zhai Q., Lu W. (2020). The protective effect of Myristica fragrans Houtt. extracts against obesity and inflammation by regulating free fatty acids metabolism in nonalcoholic fatty liver disease. Nutrients.

[b1170] Zhao R., Wang W., Zhao L., Li Z., Wang J. (2009). Effect of volatile oil from nutmeg on liver microsomal cytochrome P450 in mice. Zhongguo Zhong yao za zhi= Zhongguo Zhongyao Zazhi= China Journal of Chinese Materia. Medica.

[b1175] Zhu Y., Ouyang Z., Du H., Wang M., Wang J., Sun H. (2022). New opportunities and challenges of natural products research: When target identification meets single-cell multiomics. Acta Pharm. Sin. B.

[b1180] Zhu X., Wang Y.K., Yang X.N., Xiao X.R., Zhang T., Yang X.W. (2019). Metabolic activation of myristicin and its role in cellular toxicity. J. Agric. Food Chem..

